# Structural, Kinetic and Proteomic Characterization of Acetyl Phosphate-Dependent Bacterial Protein Acetylation

**DOI:** 10.1371/journal.pone.0094816

**Published:** 2014-04-22

**Authors:** Misty L. Kuhn, Bozena Zemaitaitis, Linda I. Hu, Alexandria Sahu, Dylan Sorensen, George Minasov, Bruno P. Lima, Michael Scholle, Milan Mrksich, Wayne F. Anderson, Bradford W. Gibson, Birgit Schilling, Alan J. Wolfe

**Affiliations:** 1 Center for Structural Genomics of Infectious Diseases, Department of Molecular Pharmacology and Biological Chemistry, Northwestern University Feinberg School of Medicine, Chicago, Illinois, United States of America; 2 Department of Microbiology and Immunology, Stritch School of Medicine, Health Sciences Division, Loyola University Chicago, Maywood, Illinois, United States of America; 3 Buck Institute for Research on Aging, Novato, California, United States of America; 4 Departments of Biomedical Engineering, Chemistry, and Cell & Molecular Biology, Northwestern University, Evanston, Illinois, United States of America; 5 Department of Pharmaceutical Chemistry, University of California, San Francisco, California, United States of America; University of Arizona, United States of America

## Abstract

The emerging view of N^ε^-lysine acetylation in eukaryotes is of a relatively abundant post-translational modification (PTM) that has a major impact on the function, structure, stability and/or location of thousands of proteins involved in diverse cellular processes. This PTM is typically considered to arise by the donation of the acetyl group from acetyl-coenzyme A (acCoA) to the ε-amino group of a lysine residue that is reversibly catalyzed by lysine acetyltransferases and deacetylases. Here, we provide genetic, mass spectrometric, biochemical and structural evidence that N^ε^-lysine acetylation is an equally abundant and important PTM in bacteria. Applying a recently developed, label-free and global mass spectrometric approach to an isogenic set of mutants, we detected acetylation of thousands of lysine residues on hundreds of *Escherichia coli* proteins that participate in diverse and often essential cellular processes, including translation, transcription and central metabolism. Many of these acetylations were regulated in an acetyl phosphate (acP)-dependent manner, providing compelling evidence for a recently reported mechanism of bacterial N^ε^-lysine acetylation. These mass spectrometric data, coupled with observations made by crystallography, biochemistry, and additional mass spectrometry showed that this acP-dependent acetylation is both non-enzymatic and specific, with specificity determined by the accessibility, reactivity and three-dimensional microenvironment of the target lysine. Crystallographic evidence shows acP can bind to proteins in active sites and cofactor binding sites, but also potentially anywhere molecules with a phosphate moiety could bind. Finally, we provide evidence that acP-dependent acetylation can impact the function of critical enzymes, including glyceraldehyde-3-phosphate dehydrogenase, triosephosphate isomerase, and RNA polymerase.

## Introduction

Post-translational modifications generate multiple protein isoforms, many of which have functional consequences. For example, N^ε^-lysine acetylation (lysine acetylation) can change protein size, conformation and/or charge, thus altering DNA binding affinity, enzymatic activity, protein stability, protein-protein interactions or protein localization [1], [2]. It is well known that eukaryotes use lysine acetylation as a primary regulatory mechanism [3]. Less well known is that lysine acetylation also occurs in bacteria. Proteomic studies of diverse bacteria have identified hundreds of acetylated proteins that function in various cellular processes [4], [5], [6], [7], [8], [9], [10], [11], [12]. Such studies have led to the supposition that protein acetylation in bacteria might play an important physiological role.

Lysine acetylation is typically thought of as the donation of an acetyl group from acetyl-coenzyme A (acCoA) to the ε-amino group of a lysine residue within a protein. It is a post-translational modification regulated by enzymes of opposing function, lysine acetyltransferases (KATs) and deacetylases (KDACs). Five protein families with KAT activity have been identified across all three domains of life [13], [14]. Of these families, the GCN5-like acetyltransferases (GNATs) are distributed most widely [15]. GNATs are ubiquitous within bacteria and can perform diverse functions. Some bacterial GNATs (e.g., Pat from *Salmonella enterica*) function as KATs, catalyzing N^ε^-acetylation of proteins [16], while others catalyze other reactions, including the acetylation of lipids or antibiotics [17], [18]. *Escherchia coli* possesses more than 25 known or putative GNATs [15]. Only one, YfiQ (a Pat paralog), has been reported to function as a KAT [19], [20]. Two types of KDACs are known: the family of NAD^+^-dependent sirtuins [21] and a family of metal-dependent enzymes [22]. Putative bacterial homologs of each KDAC family exist [23]; however, the sirtuin CobB [24] is the only known KDAC in *E. coli* [25], [26], [27], [28].

**Figure 1:**
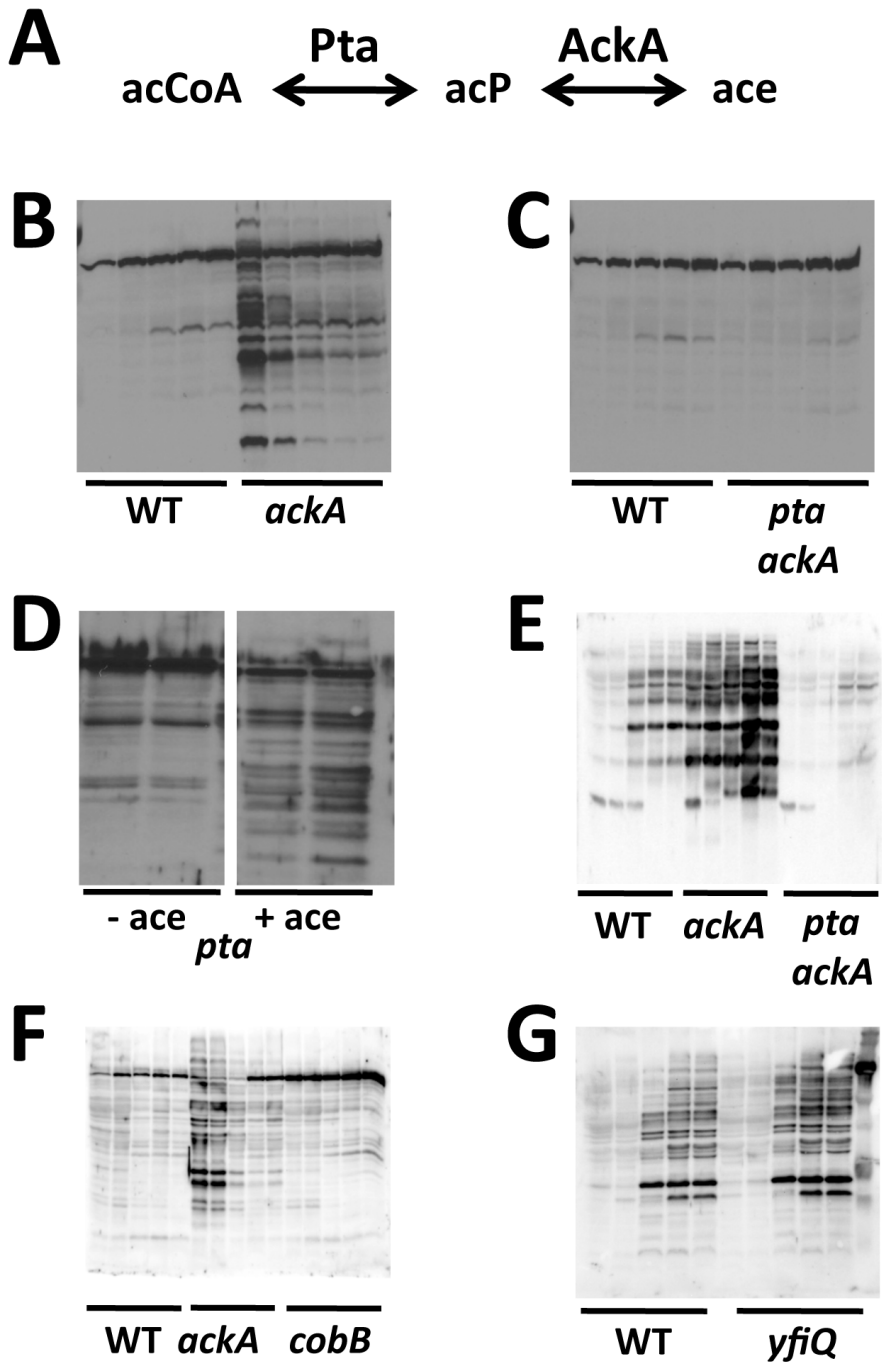
Anti-acetyllysine Western immunoblot analyses. **A**) Pta-AckA pathway schematic. **B**) *E. coli* WT (strain MG1655) and isogenic *ackA* mutant (strain AJW2012), each aerated at 37°C in TB7 and harvested at 5 time points, when the OD_610_ reached 0.5 or 1.0, and then at 8, 24 and 32 hours. **C**) *E. coli* WT (strain MG1655) and isogenic *pta ackA* mutant (strain AJW2785), each aerated at 37°C in TB7 and harvested at 5 time points, when the OD_610_ reached 0.5 or 1.0, and then at 8, 24 and 32 hours. **D**) *E. coli pta* mutant (strain AJW3699) aerated at 37°C in TB7 (-ace) or in TB7 supplemented with 10 mM acetate (+ace) and harvested at 8 and 24 hours. **E**) *E. coli* WT (strain AJW678) and isogenic *ackA* (strain AJW1939) and *pta ackA* (strain AJW2013) mutants, each aerated at 37°C in TB7 supplemented with 0.4% glucose and harvested at 5 time points, when the OD_610_ reached 0.5 or 1.0, and then at 8, 24 and 32 hours. **F**) *E. coli* WT (strain MG1655) and isogenic *ackA* (strain AJW5052) and *cobB* mutants (strain AJW5037), each aerated at 37°C in TB7 and harvested at 5 time points, when the OD_610_ reached 0.5 or 1.0, and then at 8, 24 and 32 hours. **F**) *E. coli* WT (strain MG1655) and isogenic *yfiQ* mutant (strain AJW5164), each aerated at 37°C in TB7 supplemented with 0.4% glucose and harvested at 5 time points, when the OD_610_ reached 0.5 or 1.0, and then at 8, 24 and 32 hours.

We recently reported that the acetylation patterns of RNA polymerase (RNAP) and of the response regulator and transcription factor RcsB differ substantially if purified from an acetate kinase (*ackA*) mutant rather than from its wild-type (WT) parent [29], [30], [31]. Since *ackA* mutants can accumulate acetyl phosphate (acP) [32], the high-energy intermediate of the phosphotransacetylase-acetate kinase (Pta-AckA) pathway (**Fig. 1A**) [33], [34], we hypothesized that acP could regulate the acetylation of RNAP, RcsB, and other proteins [29].

To test our hypothesis, we genetically altered the ability of cells to synthesize acP, to acetylate or deacetylate lysines, or to do both, and monitored global acetylation status by Western immunoblot analysis and quantitative label-free mass spectrometry. We used a high-throughput peptide array technology to determine if acP-dependent acetylation of susceptible lysines could occur in a non-enzymatic manner and x-ray crystallography to both confirm acP-dependent lysine acetylation of specific proteins and to examine the molecular environment of susceptible lysines. We mapped the locations of lysine residues modified in the different mutants onto previously determined protein structures in the Protein Data Bank (PDB) and performed kinetic experiments of *in vitro* acP-dependent acetylation.

We conclude that lysine acetylation is common in *E. coli*, modifying thousands of lysines on hundreds of proteins involved in diverse and often essential cellular processes. We further propose the existence of at least two distinct mechanisms for lysine acetylation: a novel acetylation mechanism that uses acP as the acetyl donor, as reported recently by Weinert and co-workers [8], as well as the conventional mechanism that requires the acCoA-dependent KAT (YfiQ). We provide validated quantitative evidence that acP-dependent acetylation is common, especially modifying proteins that function in translation, central metabolism and transcription. We provide crystallographic evidence that acP can bind to substrate and co-factor binding sites and that acP-dependent acetylation of key lysine residues of the central metabolic enzymes triosephosphate isomerase (TpiA) and glyceraldehyde-3-phosphate dehydrogenase (GapA) could alter their function. Finally, we propose that acP-dependent acetylation can be both non-enzymatic and specific, with specificity determined by the local three-dimensional (3D) environment that surrounds the substrate lysine residue, as well as by its solvent accessibility and pKa.

## Results

### acP influences lysine acetylation

To test the hypothesis that acP plays a role in lysine acetylation, we grew *E. coli* at 37°C in tryptone broth buffered at pH7 (TB7), harvested them at several intervals, and subjected the lysates to Western immunoblot analysis using an anti-acetyllysine antibody. Relative to its WT parent, we observed more acetylation in the *ackA* mutant, especially during early exponential growth (**Fig. 1B** **, Fig. S1A**) when acP concentrations are at their highest [32], [35], [36]. In contrast, lysine acetylation in cells that cannot synthesize acP (*pta ackA*) more closely resembled the WT pattern (**Fig. 1C** **, Fig. S1A**). To determine if the increased acetylation of the *ackA* mutant was due to acP, we grew a *pta* mutant in TB7 supplemented with acetate. Because this mutant retains AckA activity, it synthesizes acP from acetate independently of Pta. Since acetylation increased in an acetate-dependent manner (**Fig. 1D**), we conclude that lysine acetylation is mediated by acP itself, and not by the AckA and Pta enzymes that regulate acP levels.

We next asked if this acP-dependent acetylation required the only known KAT in *E. coli*, comparing the acetylation profiles of an *ackA yfiQ* double mutant and the *ackA* single mutant (**Fig. S1B**). Due to the lack of substantial differences in the acP-dependent acetylation profile, we conclude that YfiQ is not involved and hypothesize that acP-dependent acetylation requires a novel mechanism.

### Glucose-induced lysine acetylation depends on acP

Growth on glucose has been reported to increase acP concentrations [35] and to increase lysine acetylation [7], [8], [30]. To test if the previously reported glucose-induced acetylation requires acP, we grew WT, *ackA* and *pta ackA* cells in TB7 supplemented with 0.4% glucose and determined the protein acetylation profile by Western immunoblot analysis. Consistent with the idea that acP is a key contributor to glucose-induced acetylation, most of the signal detected from WT cells increased in cells (*ackA*) that accumulated acP and decreased in cells (*pta ackA*) that could not synthesize acP (**Fig. 1E**). Since acP-dependent acetylation was observed along the entire range of protein molecular weights, we hypothesize that acP-dependent acetylation occurs on many proteins and that acP may have a physiologically relevant role in lysine acetylation of *E. coli* proteins. This role might be larger than that of the known KDAC (CobB) and KAT (YfiQ), as the acetylation patterns in mutants lacking these enzymes closely resembled the patterns in their WT parent (**Figs. 1F and G**). Since acetylation occurs in *E. coli* cells that lack acP (*pta ackA*) (**Fig. S1C**), we conclude that there exist at least two mechanisms for lysine acetylation: one that depends on acP and one that does not.

### Mass spectrometric identification of acetylated proteins and label-free quantification of acetylation sites

To identify specific acetyllysine sites on individual acetylated proteins and to quantify sites that are clearly regulated by the acP-dependent and acP-independent mechanisms, we analyzed samples from these same strains using an in-depth mass spectrometry approach. We used a robust peptide-based affinity enrichment strategy using anti-acetyllysine antibodies [37] (1) to identify acetyllysine-containing peptides from trypsinized total protein lystates and (2) to compare the relative levels of acetyllysine-containing peptides between the WT parent and its mutants, using a recently developed label-free quantitative proteomics method referred to as ‘Skyline MS1 Filtering' [37], [38]. The label-free approach provided us with flexibility in experimental design; in particular, it allowed us to directly compare acetylation in cells grown under more physiologically relevant conditions in complex growth media, in contrast to the SILAC method used by Weinert et al. [8].

**Figure 2:**
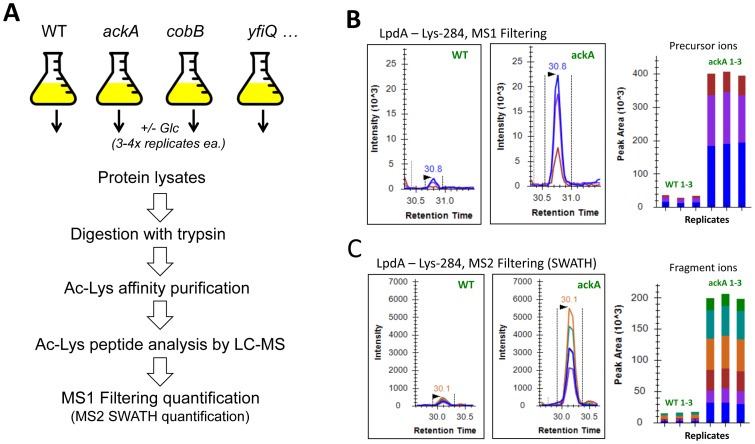
Mass spectrometric workflow. **A**) *E. coli* strains MG1655 (WT), AJW5052 (*ackA*), AJW2785 (*pta ackA*), AJW5037 (*cobB*), and AJW5164 (*yfiQ*) were aerated at 37°C in TB7 (3 independent biological replicates) or TB7 supplemented with 0.4% glucose (4 independent biological replicates) and harvested when the OD_610_ reached 1.0. For mass spectrometric analysis, the harvested cells were lysed and the protein lysates were proteolytically digested with trypsin, followed by affinity enrichment for acetyllysine (Ac-Lys)-containing peptides using a polyclonal anti-acetyllysine antibody. Enriched Ac-Lys peptides for each strain/condition were analyzed by high-resolution label-free LC-MS/MS (3 technical replicates each) for acetyl site identification, and these Ac-Lys sites were subsequently subjected to MS1- and MS2-based quantification methods. **B**) Skyline MS1 Filtering for acetylated peptide NLDAG**Kac**AGVEVDDR (^Ac^K284) obtained from dihydrolipoyl dehydrogenase (LpdA) to determine quantitative differences between the *ackA* mutant (strain AJW5052) and its WT parent (strain MG1655). MS1 ion chromatograms and corresponding peak areas demonstrating one of four biological replicates is shown for WT and *ackA* mutant (3 technical MS replicates acquired); precursor ions were extracted for M at *m/z* 750.87^++^, M+1 at *m/z* 751.37^++^, M+2 at *m/z* 751.87^++^. **C**) Independent confirmation and validation of potential candidates and sites for acetyllysine regulation using SWATH MS2 Filtering (SWATH MS2): exemplified for NLDAG**Kac**AGVEVDDR (^Ac^K284): 1 biological replicate (3 technical replicates acquired) shown with extracted ion chromatograms and corresponding peak areas for fragment ions y_12_ at *m/z* 1273.60^+^, y_11_ at *m/z* 1158.57^+^, y_10_ at *m/z* 1087.54^+^, y_8_ at *m/z* 860.41^+^, y_7_ at *m/z* 789.37^+^, and y_5_ at *m/z* 633.28^+^.

Briefly, cells from different *E. coli* strains were grown in TB7 or TB7 plus 0.4% glucose and harvested just prior to entry into stationary phase (for WT, equivalent to Fig. 1C lane 2). From either 3 or 4 biological replicates of each strain/condition, total proteins were isolated, proteolytically digested, and immunoprecipitated using a polyclonal anti-acetyllysine antibody for affinity enrichment. Subsequently, each biological replicate and all acetyllysine-enriched samples were subjected to data-dependent mass spectrometric analysis on a TripleTOF 5600 in 3 technical injection replicates (**Fig. 2A**). These mass spectrometric acquisitions were later used for label-free quantification (MS1 Filtering), which allowed pairwise comparisons of peak areas and relative abundances of acetyl sites either between strains (i.e., *ackA*/WT) or between different growth conditions (**Fig. 2B**). Lists of candidate acetyllysine sites upregulated in the *ackA* or *cobB* mutants relative to the WT were then confirmed with an independent, quantitative verification methodology referred to as SWATH-MS2, as described in detail below (**Fig. 2C**).

**Figure 3:**
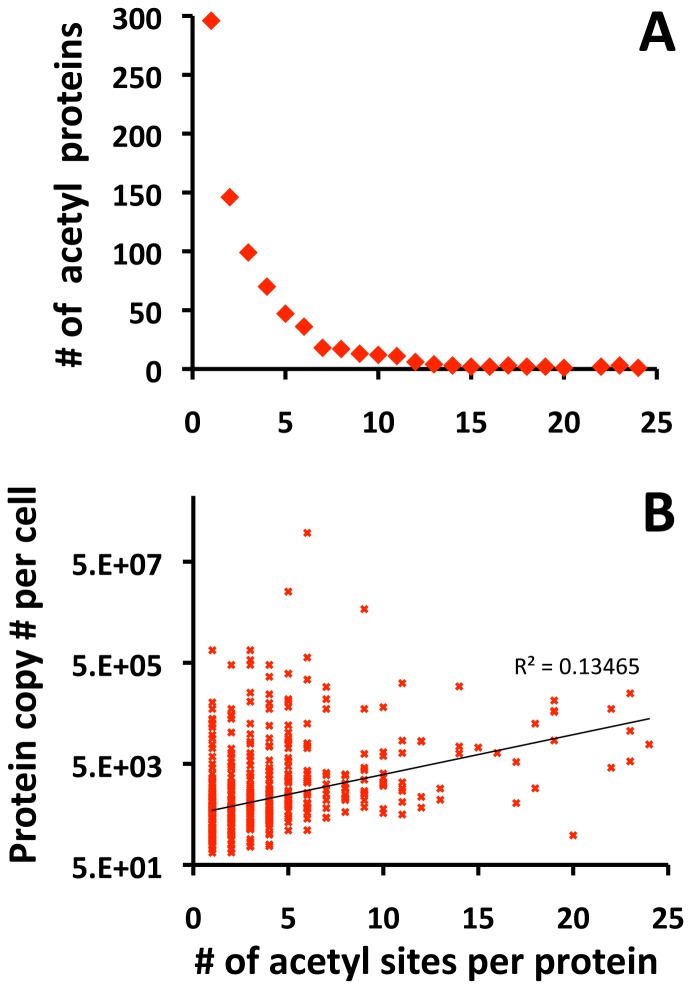
Distribution of acetylation sites in *E. coli* proteins. Mass spectrometric analysis of the strains grown in the conditions shown in Figure 2 confidently identified 2730 unique lysine acetylation sites across 806 unique acetylated *E. coli* proteins. **A**) The frequency of individual proteins relative to the number of acetyllysines per protein. **B**) The estimated protein copy number per *E. coli* cell [39] relative to the number of acetylation sites per protein. The exponential regression trendline is indicated (R^2^  =  0.13), the y-axis is presented as a logarithmic scale.

For five strains grown under two different conditions and harvested at a single time point, mass spectrometry confidently identified a total of 2730 unique acetyllysine sites on 806 acetylated proteins, as analyzed in 4 biological replicates and 3 technical replicates by LC-MS/MS (for unique acetyllysine sites, see **Tables S1-5**; for all identified acetyllysine peptides, see **Table S6-7**). The acetyllysine sites were not distributed equally among proteins. While many of the acetylated proteins were acetylated on one lysine, most were acetylated on at least two lysines and some contained as many as 24 acetyllysine sites (**Fig. 3A** **, Table S8**). This distribution (i.e, the number of acetyllysine sites per protein) did not correlate strongly with molecular weight, but did show some correlation with the total number of lysines per protein (**Fig. S2A–C, Table S8**). This distribution also showed some correlation with the estimated protein abundance [39] (**Fig. 3B** **, Table S8**), as expected of any proteomic enrichment strategy. Although it is typical for acetyllysine sites of abundant proteins to be identified frequently [37], we also observed acetyllysine sites of proteins with very low estimated protein copy numbers per cell (**Fig. 3B**).

**Figure 4:**
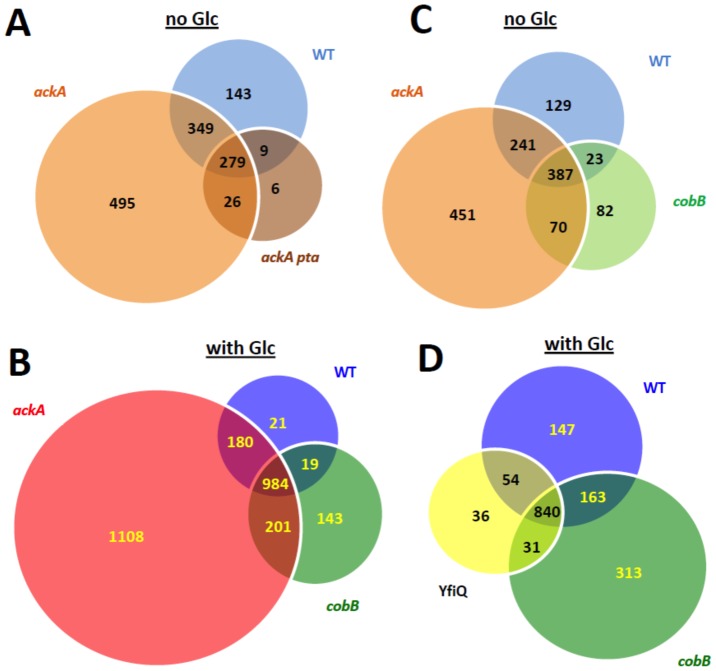
Venn diagrams showing the overlap of acetylation sites detected by mass spectrometry as described in the legend of Figure 2. **A**) Acetylation sites for WT (strain MG1655) and its isogenic *ackA* (strain AJW5052) and *ackA pta* (strain AJW2785) mutants grown in TB7. **B**) Acetylation sites for WT and its isogenic *ackA* (strain AJW5052) and *cobB* (strain AJW5037) mutants grown in TB7. **C**) Acetylation sites for WT (strain MG1655) and its isogenic *ackA* (strain AJW5052) and *cobB* (strain AJW5037) mutants grown in TB7 supplemented with 0.4% glucose. **D**) Acetylation sites for WT (strain MG1655) and its isogenic *yfiQ* (strain AJW5164) and *cobB* (strain AJW5037) mutants grown in TB7 supplemented with 0.4% glucose.

Data-dependent mass spectrometry acquisitions verified the existence of both acP-dependent and acP-independent acetylation. In the absence of glucose, we detected 780 acetylated lysine sites on 355 proteins from WT, 1149 acetylated lysine sites on 448 proteins from the *ackA* mutant and only 320 acetyl sites from 166 proteins from the *pta ackA* mutant (**Table 1** **,** **Fig. 4A**). Since *ackA* mutants accumulate acP and *pta ackA* mutants cannot synthesize acP, lysine acetylation appeared to correlate with concentration of acP. Since some acetylation was observed in the *pta ackA* mutant, we verified that some acetylation must be acP-independent. We also verified the existence of glucose-induced acetylation. For cells grown in TB7 supplemented with glucose, we detected 1204 unique acetyllysine sites on 446 proteins from WT and 2473 acetyllysine sites on 751 proteins from *ackA* (**Table 2** **,** **Fig. 4B**), numbers that are considerably larger than those observed for cells grown in the absence of glucose. In comparison, relatively small effects of both YfiQ and CobB were observed. In the absence or presence of glucose, deletion of *cobB* resulted in a small change in acetyllysine sites relative to deletion of *ackA* (**Table 1** **and** **2** **,** **Fig. 4B and C**). In the presence of glucose, relative to the WT, the number of acetyllysine sites decreased slightly in the *yfiQ* mutant and increased slightly in the *cobB* mutants (**Table 2** **,** **Fig. 4D**). Taken together, these results confirm that YfiQ and CobB affect lysine acetylation in *E. coli*, but also support our proposition that acP is a major influence on lysine acetylation.

**Table 1:** Mass Spectrometric Analysis, No glucose.

Strain	Acetylated proteins	Acetyl Lys sites
**WT**	355	780
***ackA***	448	1149
***pta ackA***	166	320
***cobB***	265	562

4 biological replicates in 3 technical MS replicates.

1388 unique acetyl Lys sites, 514 acetyl proteins.

**Table 2:** Mass Spectrometric Analysis, 0.4% Glucose.

Strain	Acetylated proteins	Acetyl Lys sites
**WT**	466	1204
***ackA***	751	2473
***cobB***	493	1347
***yfiQ***	383	961

4 biological replicates in 3 technical MS replicates.

2661 unique acetyl Lys sites, 787 acetyl proteins.

**Figure 5:**
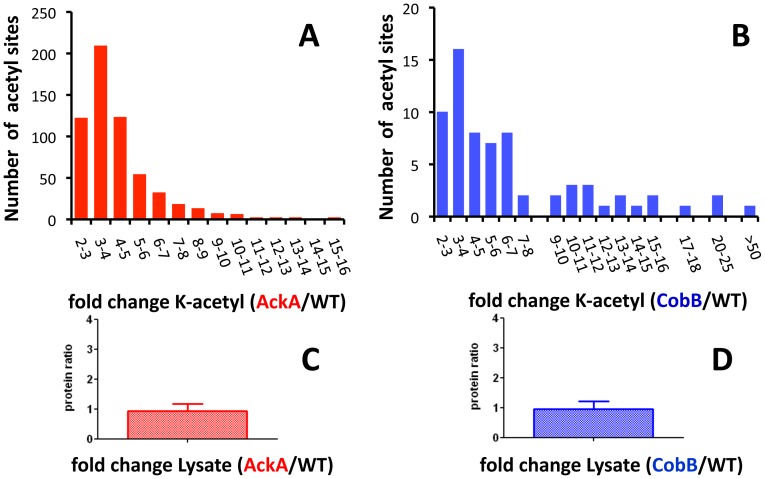
Lysines acetylated more often in the *ackA* and *cobB* mutants than in their WT parent. Quantitative mass spectrometry of acetyllysine-enriched fractions determined lysine acetylation sites that were significantly and robustly upregulated in the mutant strains compared to their WT parent across biological replicates: **A**) Fold change distribution for 592 acetylation sites from 292 acetylated proteins significantly and robustly upregulated in the *ackA* mutant relative to its WT parent. **B**) Fold change distribution for 69 acetylation sites from 51 acetylated proteins significantly and robustly upregulated in the *cobB* mutant relative to its WT parent. Corresponding protein levels were compared between the mutants and their WT parent assessing proteolytically digested total lysates: **C**) mean ratio for 292 proteins (AckA/WT) showing no protein level change with a mean ratio of 0.93. **D**) mean ratio for 51 proteins (CobB/WT) showing no protein level change with a mean ratio of 0.95.

To quantitatively compare the impacts of acP and CobB on lysine acetylation, we used MS1 Filtering [37], [38] to compare the relative changes in acetyllysine abundances in both the *ackA* and *cobB* mutants relative to the WT, when grown in the presence of glucose. For each of the four independent biological replicates, relative peak areas were extracted for each unique acetyllysine peptide in three technical replicates (**Fig. S3A**). If the mutant/WT ratio was >2 with a significance of p<0.05 in at least 3 of the acquired 4 biological replicates, then an acetyl site was considered ‘regulated’, and a mean ratio across all biological replicates was determined, as demonstrated for select examples (**Fig. S3B**). For cells grown in the presence of glucose, 592 lysines on 292 proteins in the *ackA* mutant relative to its WT parent were identified as significantly increased (**Fig. 5A** **, Table S9 and S10**). This represents 25% of the 2367 quantifiable sites for the *ackA*/WT experiment. In contrast, only 69 lysines in 51 proteins met these criteria in the *cobB* mutant (**Fig. 5B** **, Table S11 and S12**). This represents 5% of the 1392 quantifiable sites for the *cobB*/WT experiment. Thus, the number of upregulated acetyllysine sites is substantially larger in the *ackA* mutant than in the *cobB* mutant (>8-fold for acetyl sites, >5-fold for proteins). Parallel analysis of total protein lysates (i.e., without affinity enrichment), and relative quantification of unmodified peptides revealed little evidence of any differential expression changes among proteins identified as acetylated. Specifically, acetylated proteins that contained regulated acetyllysine sites showed no changes in expression levels between the either the *ackA* or *cobB* mutants and WT (**Fig. 5C and 5D** **, Table S13 and S14**), allowing us to conclude that acetylation of many lysines is dramatically upregulated in both mutants, but that the effect is much greater in the *ackA* mutant.

The fold-change distribution of all quantifiable acetyllysine sites appears to be more specific for *cobB* (**Fig. S4A, Table S15**) than for *ackA* (**Fig. S4B, Table S16**). For the *ackA* mutant, the fold-change profile for upregulated acetyllysines was broader and more global than that of the *cobB* mutant, which exhibited profile changes for 10 times fewer distinct sites. However, both mutants displayed acetyllysine site specificity, as demonstrated for a subset of acetylation proteins (**Fig. S4C and S4D**). Furthermore, the acetylation sites upregulated in the *ackA* mutant can be distinctly different from sites de-acetylated by CobB (**Fig. S4E**).

To independently validate the observed increase in acetylation of the acetyl sites regulated in the *ackA* mutant, we employed MS/MS^ALL^ with SWATH acquisition. SWATH is a comprehensive data-independent approach that is most similar to multiple reaction monitoring (MRM) assays in that it integrates sets of specific MS2 fragment ions for each targeted peptide (**Fig. 2A and 2C**; for details, see methods). However, unlike MRM, a SWATH-MS2 acquisition samples all peptide ions within a large but defined *m/z* range (400–1000 *m/z*). Of the 592 acetyllysine sites that MS1 Filtering identified as regulated by *ackA*, SWATH-MS2 was able to quantify 526, with 507 of these sites (86% of all initial 592 candidates) meeting the same threshold for upregulation (≥2-fold increase, p-value ≤0.o5) (**Fig. S5A**, **Table S17**, including all SWATH assay details, **Table S18**). For example, **Figure S5B** shows a subset of these proteins whose candidate acetylation sites were verified by both MS1 Filtering and SWATH-MS2. A similar correlation between SWATH-MS2 and MS1 Filtering was observed for potential *cobB* acetyl target sites: out of 69 candidate sites quantified by MS1 Filtering, SWATH monitored 62 and verified 59 as upregulated in the *cobB* mutant (≥2-fold increase, p-value ≤0.o5) (**Table S19 and Table S20**).

Now that our quantitative approaches had been independently validated, we used MS1 Filtering to re-examine the hypothesis that acP correlates with lysine acetylation, comparing the acetylation status of acP-sensitive acetyllysine sites in the *ackA* mutant grown in the absence of glucose to that of the same sites in the *pta ackA* mutant grown under the same conditions. The mass spectrometric results confirm the Western immunoblot analysis (**compare Fig. S6 and Table S21 to** **Figs. 1B-C and S** **1A**), showing that acetyllysine-containing peptides decreased significantly (on average 6.9-fold) in the *pta ackA* mutant relative to the *ackA* mutant.

### acP-dependent acetylation occurs in diverse cellular processes

To examine the potential biological impact of acP-dependent acetylation, we used PANTHER [40] to perform functional analyses on those lysines whose acetylation increased substantially and significantly in the *ackA* mutant relative to the WT strain. The most common molecular function of these lysines was ‘catalytic activity,’ but the functions ‘binding’ and ‘structural molecule’ activity also were common (**Fig. S7A, Table S22**). By far, the most common biological processes (**Fig. S7B, Table S23**) were metabolic in nature, i.e., ‘metabolic process’ and ‘generation of precursor metabolites and energy’, but other processes were relatively common, e.g., ‘immune system process,’ ‘response to stimulus’ and ‘transport’. Enrichment analysis by DAVID [41] using KEGG pathway (**Table 3**) terms supports the hypothesis that acP-sensitive acetylation targets central metabolism: ‘glycolysis/gluconeogenesis,’ ‘pentose phosphate pathway,’ ‘pyruvate metabolism,’ the ‘TCA cycle,’ ‘fatty acid biosynthesis’ and ‘pantothenate metabolism.’ For example, almost every protein involved in glycolysis or the TCA cycle possessed at least one acetyl site that was upregulated in the *ackA* mutant (**Fig. S8**). Many of these proteins were amongst the most acetylated, including formate acetyltransferase (PflB), phosphoglycerate kinase (Pgk), transketolase 2 (TktB), enolase (Eno), and pyruvate dehydrogenase complex E1 component (AceE), and dihydrolipoyl dehydrogenase (LpdA), a subunit of the pyruvate dehydrogenase, α-ketoglutarate dehydrogenase and glycine cleavage complexes (**Table S8**). AcP-sensitive acetylation also tends to target translation (‘ribosome’ and ‘aminoacyl-tRNA biosynthesis’), nucleotide metabolism (‘purine and pyrimidine metabolism’), and ‘RNA degradation.’ These conclusions are supported by Gene Ontology analysis (**Table S24**) and extended to include DNA-dependent transcription (GO:0006351), including all the major components of the RNAP complex (α, β, β′, and σ^70^), as well as transcription termination (GO:0006353), including NusB, NusG and Rho.

**Table 3:** Kegg pathway enrichment for 292 acetylated proteins with *ackA*-sensitive lysine acetyl sites.

Term^a^	Kegg Pathways	Count^b^	%^c^	p-value^d^	Fold Enrichment^d^	Bonferroni d ^,^ e
eck03010	Ribosome	40	13.9	4.30E-50	26.2	5.79E-47
ece00010	Glycolysis/Gluconeogenesis	17	5.9	2.95E-15	15.0	4.04E-12
ect00230	Purine metabolism	20	7.0	1.37E-13	9.3	1.85E-10
ece00030	Pentose phosphate pathway	12	4.2	1.48E-10	14.6	1.99E-07
eci00620	Pyruvate metabolism	14	4.9	2.12E-10	10.8	2.85E-07
ecq00250	Ala, Asp and Glu metabolism	11	3.8	1.38E-09	14.4	1.86E-06
ecx00020	Citrate cycle (TCA cycle)	11	3.8	3.13E-09	13.4	4.21E-06
ecd00970	Aminoacyl-tRNA biosynthesis	10	3.5	8.39E-09	14.7	1.13E-05
ece00240	Pyrimidine metabolism	12	4.2	1.80E-07	8.0	2.43E-04
ecj00061	Fatty acid biosynthesis	6	2.1	1.13E-05	17.7	0.01513
ecz03018	RNA degradation	6	2.1	2.73E-05	15.2	0.03614
ecd00640	Propanoate metabolism	7	2.4	3.12E-05	10.8	0.04112

a Kegg Pathway TERM (DAVID), a total of 332 DAVID IDs was read in.

b Number of DAVID ID's matching the specific GOTERM.

c % of count matching a specific GO category over total number of DAVID ID's entered for analysis (332).

d the *E. coli* proteome was used as background (additional analysis performed using as background ‘the total of all *E. coli* proteins identified by MS from cell lysates' resulted in the same list of enrichment categories).

e Bonferroni cutoff value of 0.05 was chosen.

### acP can function as an acetyl donor *in vitro*

While the immunological and mass spectrometric evidence clearly supports a role for acP in lysine acetylation *in vivo*, the mechanism by which acP performs this role remained unknown. We knew that acP can donate its phosphoryl group to certain response regulators [29], [31], [34]; therefore, acP could regulate protein acetylation indirectly, via a response regulator that regulates the expression or activity of a protein acetyltransferase or deacetylase. However, we currently possess no evidence for this mechanism. The acetylation profile of the *ackA yfiQ* double mutant was similar to that of an *ackA* mutant (**Fig. S1B**); thus, the only known KAT in *E. coli* cannot be intimately involved. Furthermore, deletion of *yafP*, the only putative KAT gene whose expression depends on acP [42], did not observably alter the global acetylome (data not shown).

In contrast, substantial evidence exists to support the hypothesis that acP could function directly as an acetyl donor. AcP is a high-energy compound and its chemical properties favor such a reaction. In water, it undergoes rapid hydrolysis, which increases under basic or acidic conditions and elevated temperatures [43], [44]. In the presence of Mg^2+^ or Ca^2+^ ions, acP hydrolysis increases [44], [45], because the metal ions coordinate with the phosphate to make it a better leaving group. The metal ions also neutralize phosphate's negative charge by decreasing the electrostatic repulsion. AcP also can react with imidazole to form acetylimidazole, which can hydrolyze or undergo an acyl transfer reaction to other functional groups [43], [46]. By nucleophilic attack of its carbonyl carbon, acP can chemically acetylate amines, thiols, or hydroxyl groups. In the presence of such nucleophilic reagents, the acyl transfer reaction is preferred over hydrolysis [46].

**Figure 6:**
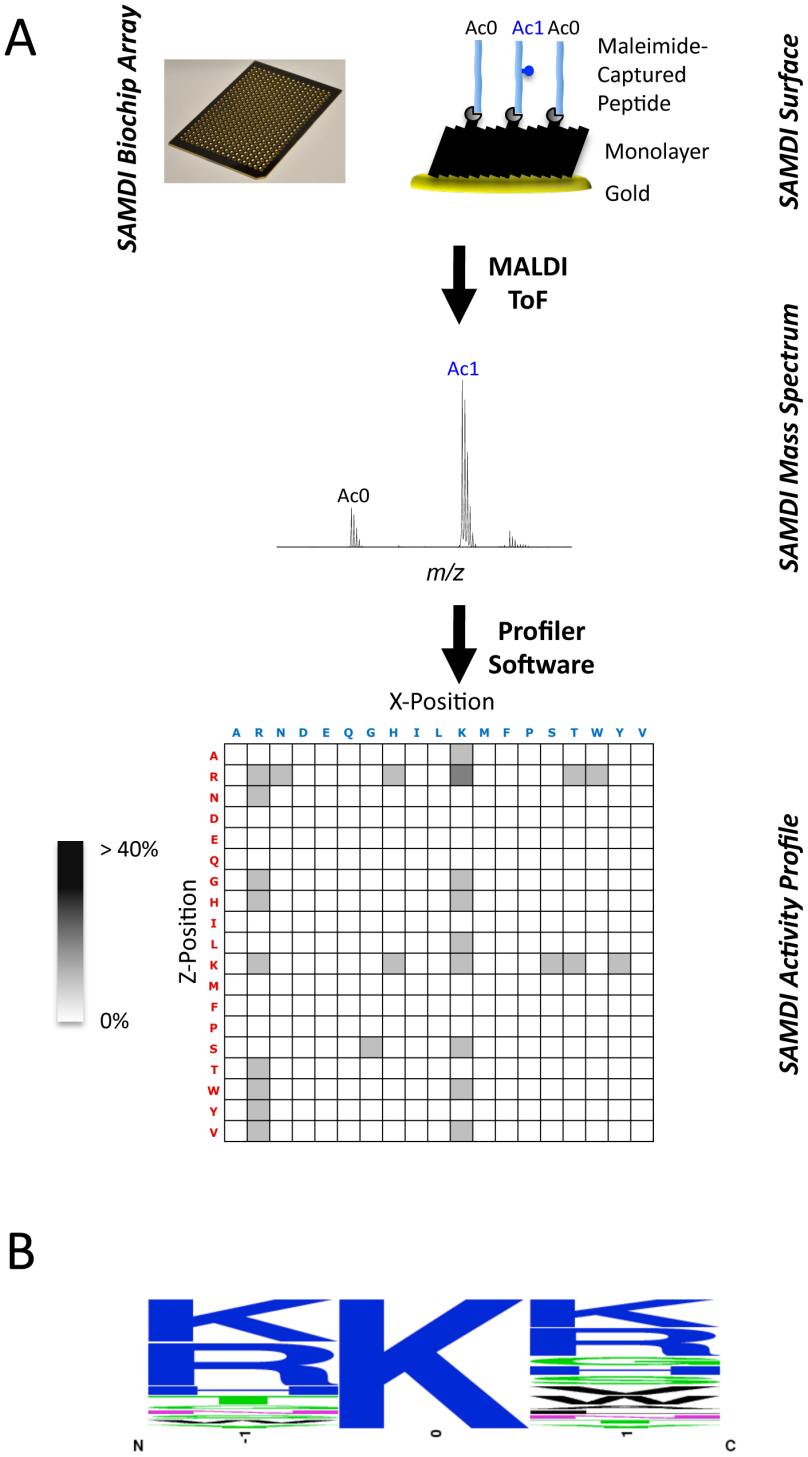
The acetylation profile of acP towards immobilized peptides containing a lysine residue. **A**) The reaction conditions contained 50 mM acP, 150 mM NaCl and 10 mM MgCl_2_. The peptide library was composed of the sequence GXKZGC, where X and Z are each standard amino acid with the exception of cysteine. Experiments were performed in duplicate and the data shown are the average of two individual trials. The shading is reflective of the percent conversion to product (acetylated peptide). **B**) Using WebLogo [50], a consensus sequence logo was generated showing the amino acid composition in the X and Z positions.

To determine if acP can react with peptides containing a lysine residue and, if so, to identify the acP chemical acetylation profile, we used a novel label-free method called SAMDI (Self-Assembled monolayers with Matrix-assisted laser Desorption-Ionization) mass spectrometry. This method monitors peptide modification of a peptide library immobilized onto a gold surface [42], [47], [48], [49], and revealed that acP can acetylate peptides non-enzymatically. At high concentrations of acP, the reaction was promiscuous and acetylated nearly every peptide. Upon the addition of salt and Mg^2+^, however, selectivity and specificity increased (data not shown). Under these conditions, the reaction was particularly favored when the acetylated lysine was adjacent to an additional positively charged residue (especially arginine (R) or lysine (K)) (**Fig. 6** **, Table S25**).

### Characterization of acP-sensitive lysines

**Figure 7:**
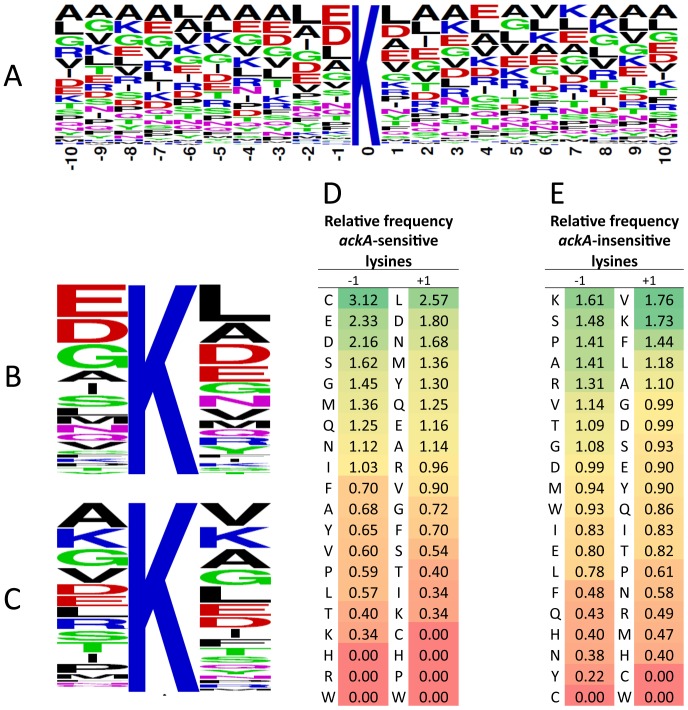
Analysis of the amino acid composition and position relative to acetylated lysines. **A**) Using WebLogo, a consensus sequence logo was generated showing the amino acid composition in positions −10 to +10 relative to 541 *ackA*-sensitive lysines (i.e., robustly, significantly and reproducibly more acetylated in the *ackA* mutant relative to WT (*ackA*/WT ratio >2 with a p-value<0.05 in at least 3 out of 4 biological replicates). Using WebLogo, consensus sequence logos were generated showing the amino acid composition in positions −1 and +1 relative to **B**) *ackA*-sensitive lysines and **C**) *ackA*-insensitive lysines from 4 proteins (LpdA, PflB, AceE, GroL). The relative frequencies were determined by comparing the frequency of each amino acid in positions -1 and +1 adjacent to the **D**) 45 *ackA*-sensitive lysines and **E**) the 131 *ackA*-insensitive lysines to the overall frequency of each amino acid in all 4 proteins (LpdA, PflB, AceE, GroL).

To see if the *in vitro* SAMDI peptide acetylation profile was recapitulated *in vivo*, we examined the protein primary sequences of the acetyl sites identified in our global mass spectrometric analysis. For this purpose, we only used the 592 acetyl sites identified as reproducibly, robustly, and significantly upregulated in the *ackA* mutant relative to its WT parent (≥2-fold increase in acetylation in at least 3 of 4 bioreplicates with a p-value<0.05). Of these 592 sites, we analyzed 541, compiling a table of neighboring amino acids from position -10 to position +10 relative to each acetyllysine. We then generated a sequence logo (**Fig. 7A** **, Table S26**), using WebLogo [50]. In contrast to the acetylation profile obtained from the *in vitro* SAMDI analysis, this analysis of acetyl sites identified by mass spectrometry of bacterial lysates revealed a high prevalence of negatively charged glutamate (E) and aspartate (D) residues in the -1 (∼30%) and +1 (∼18%) positions, adjacent to the acetyllysine residue. To determine if this prevalence was relevant to acetylation, we selected 4 proteins with multiple *ackA*-sensitive sites (LpdA, 13; PflB, 11; AceE, 10; and GroL, 11) and multiple *ackA*-insensitive sites (LpdA, 25; PflB, 39; AceE, 38; and GroL, 29) and generated sequence logos for both types of lysine (**Figs. 7B and 7C** **, Table S27**). There was a strong tendency for negatively charged residues to be located adjacent to *ackA*-sensitive lysines, with a lesser tendency relative to insensitive lysines. In contrast, positively charged residues tended to be rarely adjacent to sensitive sites, but more commonly adjacent to insensitive sites. To more rigorously test these findings, we normalized the frequency of adjacent residues to the overall frequency of those residues in these 4 proteins (**Fig. 7D and 7E**). Negatively charged residues were more likely to be in the -1 position adjacent to *ackA*-sensitive sites than to insensitive sites (p<0.009), whereas positively charged residues were more likely to be in the -1 position adjacent to insensitive sites (p<0.03) (**Table S28**). On the basis of these results, we propose that adjacent negatively charged residues play a role in acP-dependent acetylation.

**Figure 8:**
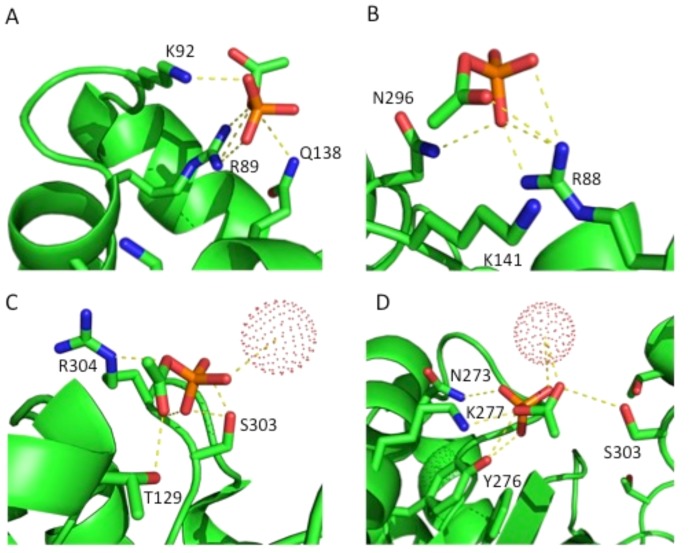
Phosphotransacetylase (Pta) from *Bacillus subtilis*, with AcP bound in four distinct sites. The crystal structure of Pta from *B. subtilis* (PDB ID: 1XCO) was used to show the locations where acP bound the protein. Yellow dashed lines highlight the polar contacts with acP, phosphate is displayed in orange, nitrogen atoms are shown in blue, and oxygen atoms are in red. Dotted spheres represent water molecules.

Since the *in vitro* and *in vivo* linear (1D) sequences did not match, we examined the 3D acP-binding environment in native, folded proteins, asking whether positively charged lysine or arginine residues were located in the vicinity of acP binding sites and/or acP-sensitive lysine residues. To begin, we analyzed the structure of phosphotransacetylase (Pta) from *Bacillus subtilis* (PDB ID: 1XCO and [51]) in complex with acP. Pta, the enzyme that catalyzes the reversible reaction that interconverts acCoA and orthophosphate to acP and CoA, crystallized as a dimer (data not shown) with four molecules of acP bound in different locations within the structure (**Fig. 8**). These acP molecules were coordinated to: (1) the ω and ω' nitrogen of R89, the ε amino group of K92, and the amide group of the side chain of Q138 (**Fig. 8A**); (2) the ω and ω' nitrogen of R88, the ε amino group of K141, and the amide group of the side chain of N296 (**Fig. 8B**); (3) a water molecule, the hydroxyl groups of the side chains of T129 and S303, and the δ amino group of R304 (**Fig. 8C**); and (4) a water molecule, the amide group of the side chain of N273, the ε amino group of K277, the hydroxyl group of the side chain of Y276 in one monomer and the hydroxyl group of the side chain of S303 from an adjacent monomer (**Fig. 8D**). Interestingly, in a study of Pta from *Methanosarcina thermophilia* (PDB ID: 2AF3), two molecules of CoA were bound to the enzyme [52] and the acP sites observed in the *Bacillus subtilis* Pta correspond to CoA pyrophosphate and ribose-3-phosphate locations. Additionally, the arginine corresponding to R304 in the third site listed above was shown to be critical for catalysis [52]. Thus, like phosphate binding sites, acP can be coordinated by positively charged residues, but also hydrogen bond donating groups such as a hydroxyl (i.e., from S, T or Y) or the side chain amide group (e.g., from Q or N).

**Figure 9:**
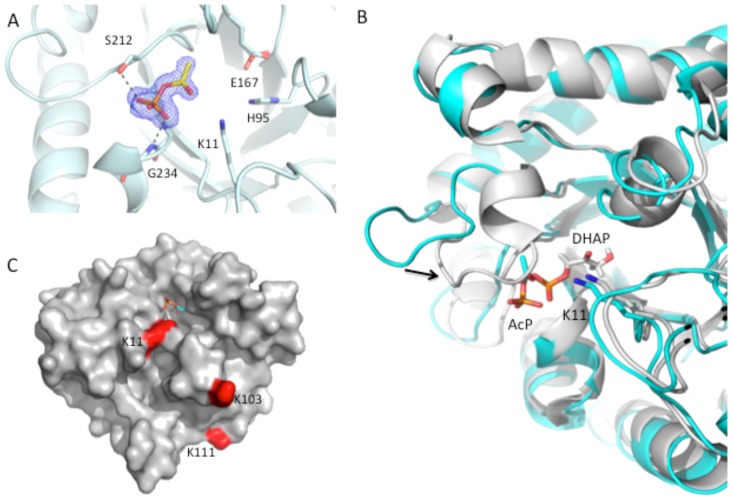
Crystal structure of *E. coli* triose phosphate isomerase (TpiA) determined in the presence and absence of acP. **A**) Cartoon and stick representation of acP bound in the active site of TpiA. The acP ligand is surrounded by the F_o_-F_c_ omit map that was contoured at the 3 sigma level. Side-chain and main-chain interactions with acP are shown as gray dashed lines. Oxygens are shown in red, nitrogens in blue, phosphate in orange, carbons of acP in yellow, and carbons of the protein in light blue. **B**) Overlay of the crystal structure of the *E. coli* acP-bound TpiA protein (PDB ID: 4MVA, cyan) with the crystal structure of TpiA from *Saccharomyces cerevisiae* (PDB ID: 1NEY, gray). The *S. cerevisiae* structure has the substrate 1,3-dihydroxyacetone phosphate (13P) bound in its active site. K11 is shown in each structure. Nitrogen atoms are blue, oxygens are red, the carbon atoms of acP are cyan and carbon atoms of 13P are gray. An arrow indicates the movement for loop closure between open (cyan) and closed (gray) forms of the protein. **C**) Surface representation of TpiA (PDB ID: 4MVA) with the locations of up-regulated acetylated lysine residues in the *ackA* mutant highlighted in red. AcP is bound in the active site.

**Figure 10:**
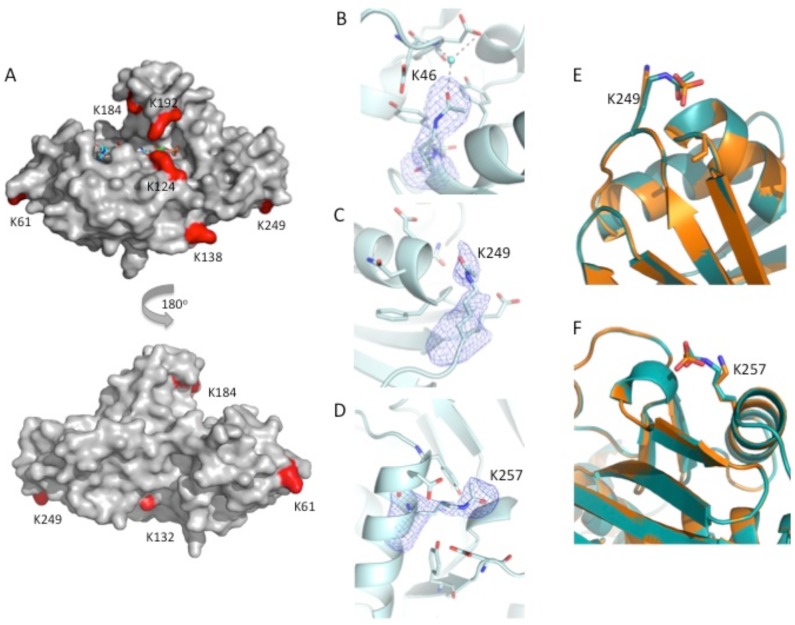
Crystal structure of glyceraldehyde-3-phosphate dehydrogenase (GapA) from *E. coli* in native (PDB ID: 1S7C) and acP-modified (PDB ID: 4MVJ) forms. **A**) Surface representation of GapA with the locations highlighted in red of up-regulated acetylated lysine residues in the *ackA* mutant as determined by mass spectrometry. Front and back views of the protein are shown and NAD^+^ (shown with sticks) is bound at the active site pocket. **B**) Electron density map surrounding acetylated K46 in the GapA crystal structure. The F_o_-F_c_ omit map in blue mesh is contoured at the 3 sigma level. Residues are shown as sticks and water is represented as a sphere. **C**) Electron density map surrounding acetylated K249 in the GapA structure. The F_o_-F_c_ omit map in blue mesh is contoured at the 2 sigma level. **D**) Electron density map surrounding acetylated K257 in the GapA structure. The F_o_-F_c_ omit map in blue mesh is contoured at the 2 sigma level. Oxygens, nitrogens, and carbons are shown in red, blue and light blue, respectively. **E, F**) Overlay of the K249 and K257 residue of GapA (PDB ID: 4MVJ) in its acetylated and non-acetylated form, respectively. Phosphate is present in the non-acetylated form and binds in the same location as the acetyl group of the acetylated residue. The protein with the non-acetylated lysine and phosphate bound is shown in orange, the protein with the acetylated lysine is in teal, oxygen atoms are in red and nitrogens are in blue.

**Figure 11:**
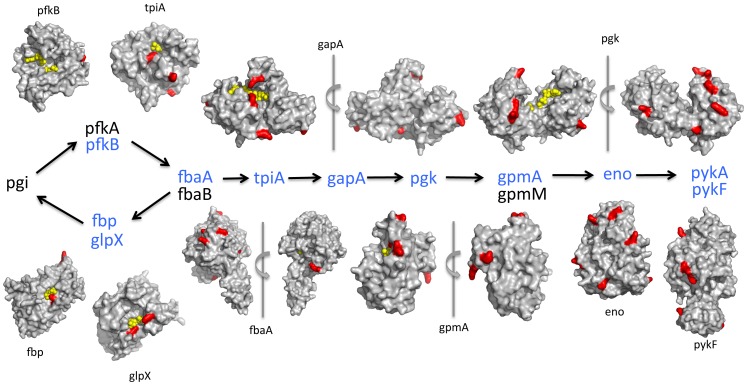
Surface representations of *E. coli* structures of glycolytic enzymes with acetylated lysines detected as upregulated in the *ackA* mutant relative to its WT parent. Proteins of the pathway that were detected as being upregulated by mass spectrometry are colored blue. The monomer of each protein, with the exception of pyruvate kinase A (PykA), is shown with a gray surface. Red patches on the structures represent upregulated acetyllysine residues. Front and back views of FbaA, GapA, Pgk, and GpmA are included and have an arrow denoting a 180° rotation of the structure. In most structures, a ligand in the active site of the protein is shown as yellow spheres.

To further characterize acP's mode of binding, we crystallized the *E. coli* enzymes TpiA (**Fig. 9**) and GapA (**Fig. 10**). Both enzymes participate in glycolysis and glucoenogenesis (**Fig. S8**): TpiA interconverts dihydroxyacetone phosphate (DHAP) and glyceraldehyde 3-phosphate (G3P), while GapA converts the G3P to 1,3-bisphosphoglycerate using NAD^+^ as a co-factor. We chose these two enzymes because our mass spectrometric results revealed that most enzymes of the glycolytic and gluconeogenic pathways possess lysine residues that are significantly more acetylated in the *ackA* mutant than in the WT parent (**Fig. 11** **, Fig. S8, Table S9**).

We determined the 3D structure of TpiA in its apo-form (PDB ID: 4K6A) and the structure obtained from similar crystals that had been quickly soaked (<30 sec) with acP (PDB ID: 4MVA). The structures showed that acP bound near K11, where the substrate DHAP is seen to bind (e.g., in PDB ID: 1NEY) (**Fig. 9A,B**). Under these conditions, K11 was not acetylated and the phosphate moiety of the acP molecule interacted with the Oγ of S212 and and the main-chain nitrogen of G234. All other interactions were water-mediated. Although quantitative mass spectrometry revealed increased *in vivo* acetylation of K103 and K111, as well as K11 (**Fig. 9C**), in the short time of the crystal soak, no acP was found in the crystal structure near either K103 or K111 and neither residue was modified (data not shown).

To obtain the structure of acP-soaked GapA (PDB ID: 4MVJ), we used a similar approach. The enzyme crystallized with four tetramers in the asymmetric unit and had either NAD^+^ or pyrophosphate bound in the NAD^+^-binding site. Not all molecules in the asymmetric unit had the same residues modified or ligands bound (**Table S29**, for more information). Quantitative mass spectrometry had shown increased acetylation of seven lysine residues, three above the active site (including K184) and four sites exterior to the active site (including K249) (**Fig. 10A**). The soaked crystals revealed that, within the short time of the crystal soak, acP-dependent acetylation had already occurred on four residues (i.e., K46, K249, K257, and K116) and three of these had well-defined electron density surrounding their acetyl groups (**Fig. 10B-D**). For these three lysines, no other positively charged residues were present in the surrounding environment, though they were located near carboxylates. Because the crystallographic experiments reveal acP binding and lysine acetylations that have reached significant fractional occupancy in the crystal environment in less than one minute, whereas the mass spectrometry data defines the fold increase in acetylation state, it should not be surprising that the results do not correspond precisely.

Taken together, these crystallographic results confirm the hypothesis that acP can acetylate lysine residues in the absence of an acetyltransferase. They also demonstrate that acP binding can be coordinated by functional groups with positive charges, hydroxyls, amides, or combinations of each, while acetylated lysines frequently also have carboxyl groups in the immediate neighborhood.

### *In vitro* acetylation of LpdA and RNAP

**Figure 12:**
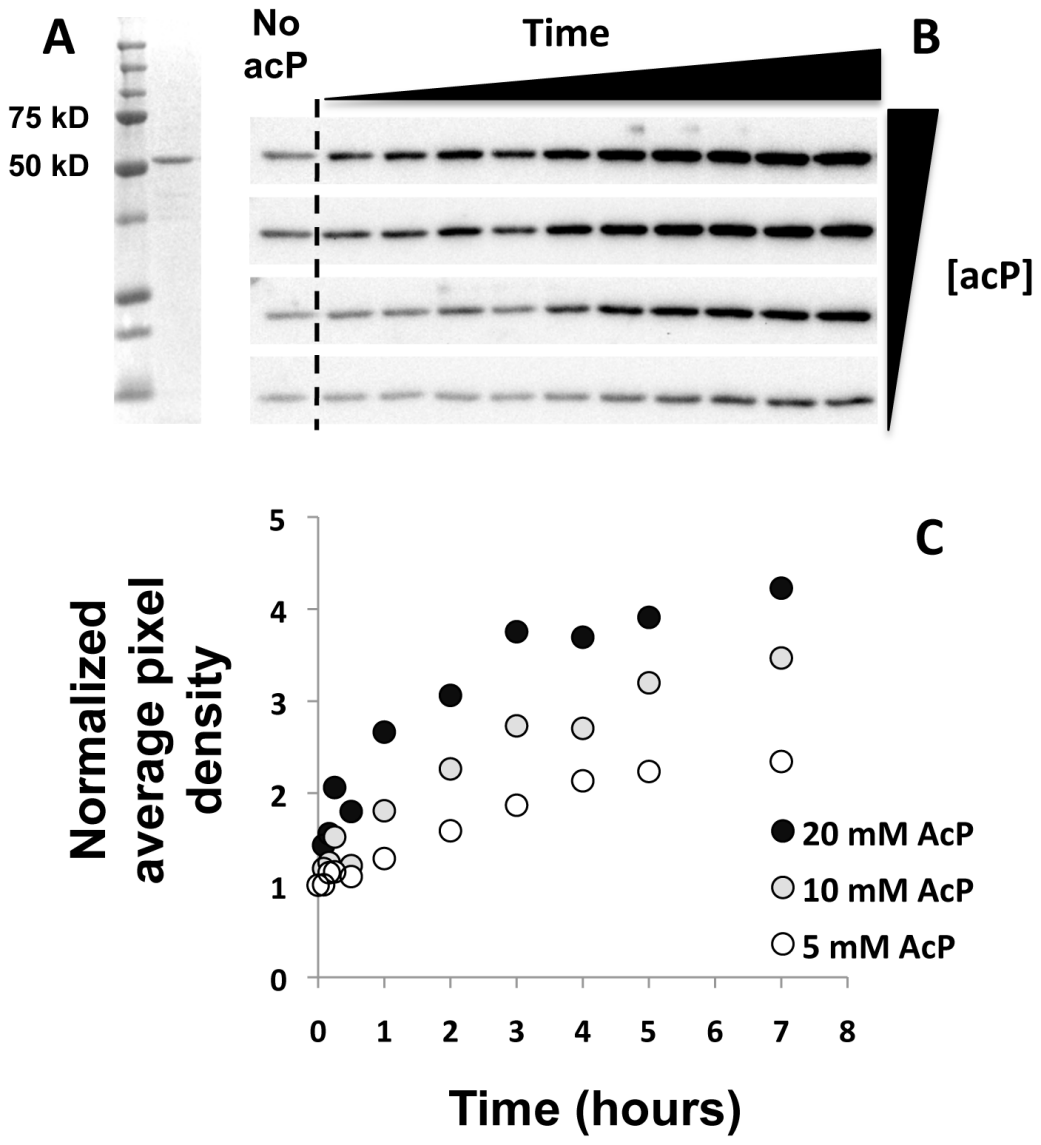
*In vitro* acetylation of LpdA using acP as the acetyl group donor is sensitive to acP concentration and time of incubation. Coomassie stain of SDS-polyacrylamide gel and anti-acetyllysine Western immunoblot analysis of acP (5, 10, 15, 20 mM) incubated with 1.25 µM LpdA for various lengths of time (5, 10, 15, and 30 min, 1, 2, 3, 4, 5, and 7 hours) at 37°C. Acetylation signals were quantified using AlphaView and normalized to the signal in the absence of acP.

While crystallography showed that acP could be an acetyl group donor to proteins *in vitro*, it could not reveal the kinetics of that reaction. To obtain kinetic information, we used the pyruvate dehydrogenase subunit LpdA because mass spectrometry analysis of the *E. coli* lysates showed that it was highly acetylated in the *ackA* mutant relative to the WT parent (**Table S9**). Indeed, when incubated with acP, purified LpdA became acetylated in a dose- and time-dependent manner (**Fig. 12**). The most obvious increases in overall acetylation occurred in the presence of acP concentrations (10 and 20 mM) within the endogenous range detected in an *ackA* mutant [53], [54]. Using these acP concentrations, we monitored relative acetylation of specific LpdA lysine residues over time, performing MS1 Filtering quantitation on two technical mass spectrometric replicates of two independent incubation replicates. This analysis confirmed the overall increase in acetylation observed by Western immunoblot analysis (**compare Fig. S9A to** **Fig. 12**) and revealed specific relative increases in acetylation of 6 and 11 lysine residues when LpdA was incubated with 10 and 20 mM acP, respectively (**Fig. S9A and B; Tables S30 and S31**). These acetylated sites were similar to those detected *in vivo*, suggesting that acP could regulate some LpdA acetylation *in vivo*.

**Figure 13:**
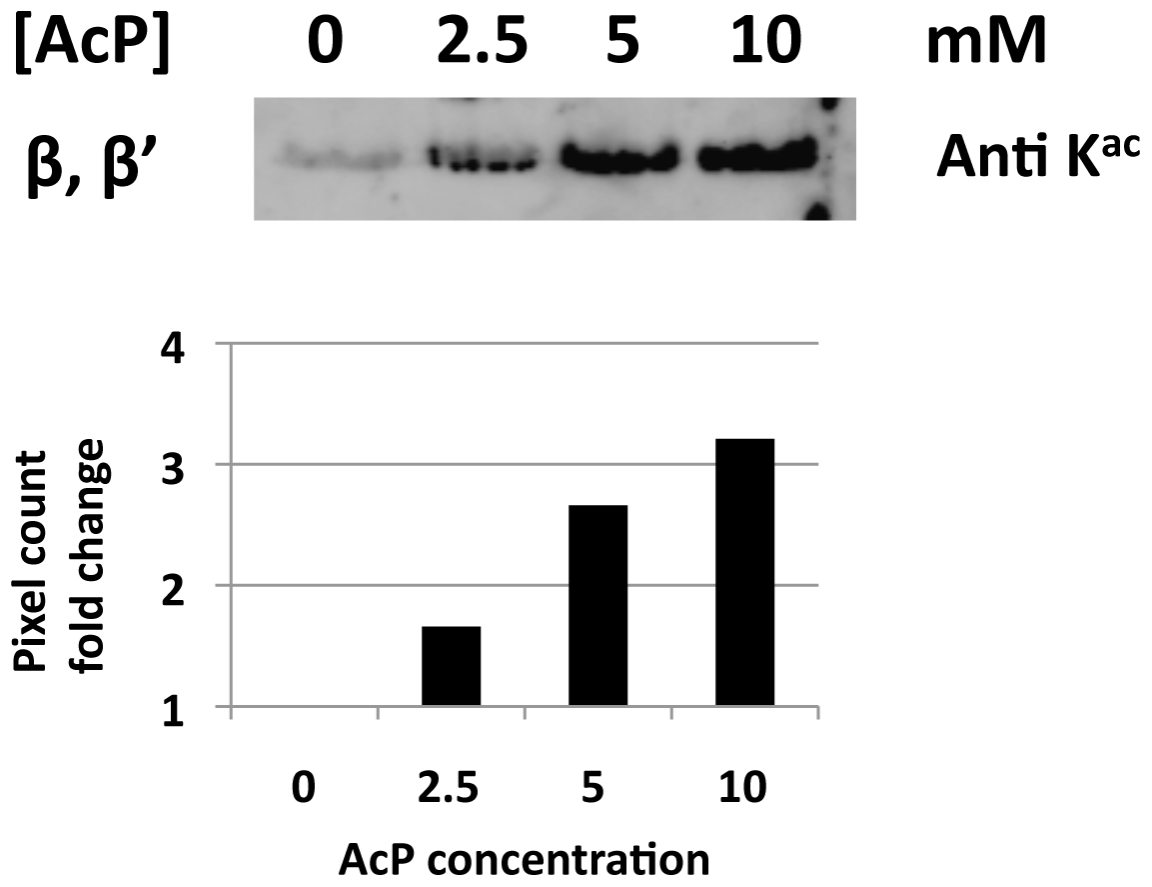
*In vitro* acetylation of RNAP with AcP. Purified RNAP σ^70^ holoenzyme was incubated with increasing concentration of acP for 1 hour at 30°C. After incubation, proteins were resolved by SDS-PAGE and transferred to a PVDF membrane. Protein acetylation was detected using a cocktail of two polyclonal anti-acetyllysine antibodies was used (Cell Signaling Technology) at a 1∶200 dilution and (ImmuneChem) at a 1∶500 dilution. Acetylation signals were quantified using AlphaView and normalized to the signal in the absence of acP.

Because we had previously published on the acetylation of RNAP [30], [31] and because we observed substantially increased *in vivo* acetylation of several RNAP lysine residues in the *ackA* mutant relative to the WT parent, purified RNAP was incubated with a range of acP concentrations for 30 minutes. By Western immunoblot analysis, we observed acetylation of the subunits β (RpoB) and β′ (RpoA) (**Fig. 13**). Using MS1 Filtering (**Fig. S10, Table S32**), significant relative increases in acetylation were observed for K297 of RpoA, for K1065 of RpoB, and for K493 and K593 of RpoD (the σ^70^ subunit). On the basis of these data and those obtained with LpdA, we conclude that acP-dependent acetylation depends on both acP concentration and duration of incubation.

For particularly reactive lysine residues of LpdA and RNAP, we assessed the correlation of their acetylation with their pKa and surface accessibility (**Tables S33–36**). Typically, the surface accessibility of the acetylated lysine was more important than its pKa. One interesting exception was K1065 of RpoB, whose acetylation status increased dramatically (14.8 fold) upon acP incubation. Although K1065 is not exposed to the surface, it has a somewhat lower calculated pKa value (9.2) than other acetylated RpoB lysines. This lower pKa, combined with the identity and location of neighboring residues, could explain the elevated reactivity of K1065.

## Discussion

### Lysine acetylation is abundant in *Escherichia coli*

We used a label-free quantitative mass spectrometric workflow to provide comprehensive, reproducible, and validated evidence that lysine acetylation is abundant in *E. coli*. This label-free approach, MS1 Filtering followed by SWATH-MS2 validation, allowed us the flexibility to analyze cells in tryptone broth, a complex mixture of amino acids, short peptides, nucleotides, and vitamins that forms the basis for many of the most common media, including Luria broth. This approach in general will permit global mass spectrometric analyses of organisms that must be grown in complex media or feedstocks, such as fastidious pathogens (e.g., *Mycobacterium tuberculosis, Haemophilus influenzae* or *Neisseria meningitidis*), industrial strains (e.g., those being developed for biofuel production), or bacteria grown in tissue culture to test associations with eukaryotic cells (e.g., urinary microbiota in association with human urothelium).

Using our label-free approach, we detected 2730 unique acetyllysine sites on 806 acetylated proteins from an isogenic set of strains harvested at a single time point (**Tables 1** **and** **2** **,** **Fig. 4** **, Tables S1–S5)**. The detected acetyllysine sites were not distributed equally. Most of the acetylated proteins contained one or two acetylated lysines, but some contained as many as 24 (**Fig. 3A** **, Table S8**). Whereas the distribution of acetyllysines did not correlate with molecular weight, it trended with the total number of lysines per protein (**Fig. S2**) and with estimated protein abundance (**Fig. 3B**). The dynamic range of detection of acetylated sites covered about 7 orders of magnitude, identifying acetylated lysines from proteins with very low, as well as very high, estimated protein copy numbers per cell (**Fig. 3B** **, Table S8**). Since the TripleTOF 5600 mass spectrometer when combined with HPLC separation typically shows a dynamic range of 4-5 orders of magnitude, we conclude that expansion of the dynamic range was achieved by our acetyl-enrichment strategy.

Our quantitative mass spectrometric assessment of lysine acetylation was performed in a label free environment simulating physiological conditions that could not have been achieved easily using metabolic SILAC labeling strategies, which require specific growth media and strain manipulations. To assess both technical and biological variance, we performed 3 technical replicates on either 3 or 4 independent biological replicates. Strict statistical requirements were applied to identify acetylation sites that were upregulated in mutants relative to their WT parent, arriving at final candidate lists of 592 and 69 lysines that were significantly more acetylated in the *ackA* or *cobB* mutants, respectively. Our use of an MRM-like data-independent method (SWATH-MS2) confirmed the majority of the acetylated candidates (86% and 86% for the *ackA*- and *cobB*-sensitive sites, respectively). An added advantage of SWATH-MS2, a quantitative ‘targeted proteomics’ method [55], centers on its ability to monitor many sites with a quantitative data-independent acquisition manner. As such, it is a high-throughput assay that requires little prior assay development. To our knowledge, these data are the most statistically rigorous and validated quantitative analysis of global acetylation in bacteria.

### Exposure to glucose enhances lysine acetylation

By buffering the complex medium, we eliminated pH effects caused by metabolism of amino acids (alkalinization) and/or glucose (acidification). As growth under these conditions did not depend on the glucose but rather on the underlying tryptone, this allowed us to rigorously test the proposal that exposure to glucose increases lysine acetylation [7], [8], [30] in a physiologically relevant condition (i.e., natural transition into stationary phase) and to identify lysines that become acetylated in the additional presence of glucose in the medium. Using both immunological and mass spectrometric analyses on our isogenic set of strains, we verified that glucose does indeed increase global protein acetylation, about two-fold under the tested growth conditions (**Tables 1** **and** **2** **,** **Fig. 4**).

The mechanism by which glucose increases lysine acetylation remains unknown; however, it may involve the acCoA-to-CoA ratio. We recently reported that glucose induces transcription from the envelope stress response promoter *cpxP*, that this effect can be mimicked by growth of these cells in the presence of the CobB inhibitor nicotinamide, that glucose-induced *cpxP* transcription depends on K298 of the C-terminal domain of the α subunit (α-CTD) of RNAP, and that K298 is acetylated under these conditions. Intriguingly, glucose-induced *cpxP* transcription is dampened by three distinct genetic manipulations that reduce the acCoA-to-CoA ratio [30]. Additional evidence suggests that certain other glucose-induced promoters behave similarly (L. I. Hu and A. J. Wolfe, unpublished data).

One possible consequence of a high acCoA-to-CoA ratio is the accumulation of acP. Indeed, we showed that the glucose effect upon lysine acetylation was enhanced when acP levels were high (*ackA*), but nearly abolished when acP was absent (*pta ackA*) (**Fig. 1E**). We conclude that glucose-dependent enhancement of lysine acetylation requires acP. We further propose that simultaneous exposure to both mixed amino acids and glucose (e.g., in the urine of a diabetic patient) may be a physiologically relevant condition under which the previously reported acP-dependent acetylation [8] might play a key role.

### acP influences lysine acetylation of proteins in diverse cellular processes

Our immunological analyses of isogenic sets of strains provided definitive genetic evidence that much, but not all, of lysine acetylation in *E. coli* is mediated by acP (**Fig. 1**), that this effect behavior is not strain-specific (**Fig. S1A**), and that acP-dependent acetylation does not depend on YfiQ (**Fig. S1B**). These results strengthen and extend the proposals of Weinert and co-workers [8].

The glucose-induced acP-dependent acetylation identified by label-free quantitation (**Fig. 5** **, Table S9**) was enriched in essential processes such as translation, central metabolism and transcription (**Table 3** **, Fig. S8, and Table S24**). This enrichment does not seem to be associated with the glucose, but rather with acP, as a similar pattern was observed in the 84 acP-dependent acetyllysines that were more acetylated in *ackA* cells grown in the absence of glucose compared to the *pta ackA* cells grown under the same conditions (**Fig. S6 and Table S21**).

Previous reports highlighted the tendency of bacterial proteins involved in translation and central metabolism to be acetylated [4], [5], [7], [8], [9], [10], [11], [12]. To our knowledge, however, ours is the first attempt to perform pathway enrichment analysis, which reveals that these processes are particularly sensitive to acP-dependent acetylation. An obvious question is why? For central metabolism, the answer might be as simple as providing a mechanism by which cells respond to the overflow metabolism that occurs when the carbon flux exceeds the capacity of central metabolic pathways, e.g. the TCA cycle. One major consequence of overflow metabolism is the conversion of acCoA to acP, accumulation of which would cause acetylation of the glycolytic enzymes that convert carbon to acCoA and thus to acP [33]. A similar rationale might be applied to the proteins of translation. WT cells that accumulate acP are growing rapidly. Perhaps acP-dependent acetylation of ribosomal proteins and other translation-associated proteins alters their function to accommodate more rapid growth.

### acP-dependent lysine acetylation can be non-enzymatic

Since it was clear that acP influenced lysine acetylation, we sought the mechanism by which it performs this function. We knew that acP can donate its phosphoryl group to certain response regulators [29], [31], [34], but we found no evidence that acP regulates lysine acetylation by controlling the expression of *yafP*, the only putative KAT gene whose expression depends on acP [42]. In contrast, we obtained substantial evidence to support the hypothesis that acP could function directly as an acetyl donor, as proposed [8], [56]. *In vitro*, acP acetylated GapA (**Fig. 10**), LpdA (**Fig. 12**), and RNAP (**Fig. 13**). Thus, acP can clearly function as an acetyl donor for lysine acetylation. Furthermore, the process appears to be non-enzymatic in nature as (*i*) acP acetylated peptides *in vitro* (**Fig. 6**), (*ii*) acP-dependent acetylation of GapA occurred within the crystal lattice, and (*iii*) a kinetic analysis of acP-dependent acetylation of purified LpdA showed the reaction to be slow and non-saturating (**Fig. 12** **, Figs. S9 and S10, Tables S30 and S31** and data not shown), which are characteristics of non-enzymatic reactions [57]. Thus, we conclude that acP-dependent acetylation does not require an acetyltransferase. We further conclude that acP-dependent acetylation must be catalyzed by residues that surround the lysine substrate (see below). Whether acP-dependent acetylation occurs non-enzymatically *in vivo* remains to be determined. However, the lysines of GapA, LpdA and RNAP that became acetylated *in vitro* closely paralleled those identified as upregulated in the *ackA* mutant (compare **Fig. 10** **, Fig. S9**, and **Table S30–S32** to **Table S9**). Moreover, there is precedence for non-enzymatic modifications; for example, acCoA-dependent acetylation and succinyl-CoA-dependent succinylation in mitochondria [58]. Indeed, some non-enzymatic modifications have been implicated in human disease processes (e.g., glycation in diabetes) [59].

### acP-dependent lysine acetylation can influence protein function

The effect of acP-dependent acetylation on cellular processes remains to be fully explored; however, some evidence exists. First, the most common molecular function of acP-sensitive lysines was ‘catalytic activity’ (**Fig. S7A, Table S22**). Indeed, four of the ten central metabolic enzymes whose structures were analyzed (**Fig. 11**) have lysines within their active sites with significantly increased acetylation in the *ackA* mutant relative to the WT parent (**Table S9**); acetylation of these residues would be expected to reduce enzymatic activity. For example, the soaking of TpiA crystals with acP revealed an acP molecule bound near K11 in both active sites, in a position similar to that of bound DHAP. Thus, the binding of acP or the modification of this residue would be expected to inhibit binding of the substrate and thus TpiA activity. If acP were to acetylate K11, a site identified in our mass spectrometry experiments (**Table S9**), it would likely inactivate the enzyme. This expectation is based on the observation that the cationic side chain of K11 in yeast TpiA is critical for catalysis because it stabilizes the transition state of the reaction [60]. Removing this charge in the yeast enzyme by substituting it with a glycine residue effectively rendered the enzyme inactive [60], and caused a decrease in activity of four-orders of magnitude for isomerization of G3P and a nearly 50-fold decrease in affinity for G3P [60].

Although acP was bound in the active site of TpiA, it was not oriented properly for efficient nucleophilic attack on the carbonyl carbon (**Fig. 9B**). Unlike other structures of TpiA with the substrate DHAP (PDB ID: 1NEY), the transition-state analog 2-phosphoglycolic acid (PDB IDs: 2BTM, 1AW1), or orthophosphate (PDB ID: 1AG1) bound in the active site, our acP-bound structure did not efficiently trigger lid-closure of Ω loop 6 (comprised of residues 168-178) (**Fig. 9B**). Our crystal structure showed alternative conformations for this loop, likely due to crystal packing and contacts between different molecules of the asymmetric unit, especially the main chain nitrogen of T174 of one monomer and the main chain carbonyl oxygen of K152 of another monomer. Such effects likely contributed to the improper orientation of acP, prohibiting efficient acetyl transfer to K11.

Acetylation also could inhibit enzyme activity by interfering with the binding of co-factors, allosteric effectors or protein-protein interactions. For example, in the case of GapA, acetylation of K183 of the yeast, sturgeon muscle, and rabbit muscle enzymes (which is homologous to *E. coli* K184) decreases the enzyme's affinity for NAD^+^ [61], [62], resulting in a loss of enzymatic activity [61], possibly through altered inter-subunit interactions. This phenomenon could be more general, as many of the *ackA* upregulated acetyllysines of the structurally analyzed metabolic enzymes (**Table S9,** **Fig. 11**) were located exterior to the active site.

Acetylation could impact protein-nucleic acid and protein-protein interactions. Glucose induces *cpxP* transcription in WT cells in a manner that depends on lysine acetylation, likely via K298 of the α-CTD of RNAP, which is acetylated under these conditions [30]. In an *ackA* mutant, however, *cpxP* transcription is inhibited in a K291-dependent manner, a genetic mimic of a constitutively acetylated K291 (K291Q) causes inhibition in the WT parent [31], and K291, but not K298, is upregulated about three-fold in *ackA* mutants grown in the presence of glucose (**Table S9**) [31]. K298 is involved in DNA-binding [63], while K291 is known to be involved in at least one protein-protein interaction [64], and thus could interact with CpxR, the response regulator and transcription factor required for *cpxP* transcription [65]. That acP-dependent acetylation could influence protein-protein interactions is supported by the work of Eisenbach and colleagues, who have shown that acetylation of CheY diminishes its affinity for its 3 interaction partners, CheA, FliM, and CheZ [66].

An intriguing possibility is that acP, as a very small molecule, could gain access to interior surfaces of proteins that enzymes cannot; for example, the interior of a large complex (e.g., the ribosome or RNAP) where the active sites are often located. Indeed, K1065 of the β subunit sits inside the RNAP complex where it helps align the first ribonucleotide of every nascent transcript [67]. Acetylation and thus neutralization of this key lysine would be expected to render the acetylated complex inactive. Because a deacetylase would be unable to gain access to the interior of RNAP, this inaction almost certainly would be permanent. Since RNAP is a scarce resource [68], acetylation-mediated inactivation of even a small fraction of RNAP could have a large global impact. In the *ackA* mutant, K1065 was upregulated ∼3.5-fold.

Like K1065 of the RNAP β subunit, other lysines located in active sites of proteins that bind a phosphorylated substrate could be acetylated by acP. Indeed, acetylation of a strategically located lysine could influence the binding of not only substrates, but also of co-factors and allosteric effectors. The likelihood that such an acetylation would occur should depend upon competition with the concentration of those substrates, co-factors, or effectors, which could protect against modification. Thus, detection of these acetylations should vary with the method of detection (e.g., *in vitro* versus *in vivo*). This might explain why, of the glycolytic enzymes that we analyzed, only a handful of the detected acetylated lysines were near the active sites.

Finally, since acP contains a phosphoryl group, its ability to compete with phosphorylated substrates, co-factors, or effectors would not actually require modification; it could simply bind to sites normally occupied by phosphorylated substrates, effectors, or cofactors and sterically hinder their binding (e.g., **Fig. 9B** **,** **10E, F**).

### Defining the characteristics of an acP-sensitive lysine acetylation site

Although acP-dependent acetylation seemed to have a broader set of targets and appeared to generally be less specific than CobB-sensitive acetylation (**Fig. S4A and S4B**), acP-dependent acetylation showed high specificity for selected proteins and for selected lysines (**Fig. S4D**) that could be differentiated from those that were sensitive to CobB (**Fig. S4E**). Biochemical, bioinformatic and crystallographic efforts to identify the characteristics of an acP-acetylatable lysine revealed a tendency toward a surface-exposure microenvironment enriched for residues with positive charges, hydroxyls or amides, but often with an adjacent negatively charged residue. In contrast to previous reports, we extended our analysis to the structures of proteins that mass spectrometry had detected as acetylated and found a uniqe three-dimensional microenvironment (**Fig. 8** **–** **10**).

It would appear that such a micoenvironment requires two properties: an affinity for acP and a way to enhance the reactivity of the lysine. The affinity requirement appears to be satisfied by the formation of salt bridges between the negatively charged phosphoryl group of acP and positively charged residues of the protein to be acetylated. An alternative or additional contribution is from hydrogen bonds between the phosphoryl group and hydrogen bond donors such as hydroxyl and/or amide functional groups that surround the lysine. The enhancement of the reactivity of the lysine might be satisfied by an adjacent negatively charged residue, an observation reported recently for acetyllysines in *Thermus thermophilus* [6]. We base this concept upon studies of acetyl transfer by members of different KAT families [69], [70], [71]. Acetylation is proposed to occur by direct transfer of an acetyl group onto the substrate following formation a ternary complex between the substrate, acCoA, and the KAT [69], [71], [72]. However, acetyl transfer requires deprotonation of the lysine. At high pH, protonation can occur non-enzymatically. At neutral pH, however, the epsilon amino groups of most lysine side chains remain largely protonated due to their high pKa's and thus are not good nucleophiles. Several mechanisms for catalytic deprotonation have been proposed. For example, the KAT can use a negatively charged residue (i.e., D or E) as a catalytic general base to deprotonate lysine and thus activate it for acetylation. Structural and mutational analyses of the eukaryotic KAT Gcn5 protein suggest that deprotonation occurs via a conserved glutamate residue (E173) [73], [74]. Mutational analysis of E173 determined that this residue was not involved in binding to either acCoA or its histone substrate. Because E173 was not required for catalysis at high pH, a condition that non-enzymatically deprotonates lysine, the authors proposed that E173 functions as a general base to deprotonate lysine for nucleophilic attack of acCoA at neutral pH. This mechanism appears to be conserved as catalytic activity for the eukaryotic KAT Rv0998 depends on E235, which is positioned similarly to the conserved E173 of Gcn5 [75].

Several divergent eukaryotic KATs possess residues required for catalytic activity that could act as general bases to deprotonate the lysine substrate [69], [73], [75], [76]. For example, Naa50p-dependent acetylation depends on tyrosine (Y73) and histidine (H112), residues proposed to act as general bases [75]. Finally, instead of a catalytic general base, some KATs have a series of residues that may form a water channel or a ‘proton wire’ that could shuttle protons from the active site to internal proton acceptors or to the solvent [70], [77], [78]. For acP-dependent acetylation, similar mechanisms could operate.

### Concluding Remarks

We conclude that *E. coli* possesses two mechanisms for lysine acetylation: the conventional KAT- and acCoA-dependent mechanism and the acP-dependent mechanism described here. The full extent of the YfiQ-dependent acetylation remains to be determined. While some have suggested diverse roles [7], [20], the only well-established role is to acetylate acetyl-CoA synthetase and thus regulate its activity [16]. In contrast, the effect of non-enzymatic acetylation of proteins by acP appears to be quite extensive. Concerning this novel mechanism of lysine acetylation, several questions remain unanswered. How does the cell cope with increases in acP-dependent protein acetylation? Does acP-dependent acetylation simply accumulate over time or is the process regulated? For example, CobB could recognize and deacetylate acetyllysines acetylated by acP. This mechanism is suggested by the sensitivity of some sites (e.g., K154 of RcsB) to both acP and CobB, but other acP-sensitive sites seem to be unrecognized by CobB. What is the effect of acP-acetylation on protein function? Our data suggest that it can inhibit enzymatic activity by acetylating key lysine residues in active sites, allosteric sites, or cofactor-binding sites. But, non-enzymatic acetylation by acP also could influence protein stability, oligomerization and protein-protein interactions, protein-nucleic acid interactions, protein translocation to various regions of the cell, cellular signaling mechanisms, or even host-pathogen interactions. What is the stoichiometry of acP-dependent acetylation? The fraction that any given lysine is acetylated at any given moment should determine the degree to which that modification alters the cellular process in which it functions. For rate-limiting enzymes or proteins in scarce supply, the stoichiometry should be crucial. Finally, how does the full extent of acP-dependent acetylation influence cellular physiology? Mutants of the Pta-AckA pathway exhibit pleiotropic phenotypes [33]. How many of these phenoytpes result from dysregulated acP-dependent acetylation? Since acP plays many roles (e.g., generating ATP, phosphorylating certain RRs and acetylating proteins) [33], [34], the answer to this question will require a series of precise genetic dissections.

## Materials and Methods

### Materials

Chemical reagents and media components were purchased from Fisher Scientific (Pittsburgh, PA), Sigma Chemical Company (St Louis, MO), Difco (Sparks, MD), J.T. Baker (Center Valley, PA), Cardinal Health (Dublin, OH) and United States Biochemical (Cleveland, OH). Nitrocellulose was purchased from BioRad (Germany). HPLC solvents including acetonitrile and water were obtained from Burdick & Jackson (Muskegon, MI). Reagents for protein chemistry including iodoacetamide, dithiothreitol (DTT), ammonium bicarbonate and formic acid were purchased from Sigma Aldrich (St. Louis, MO). HLB Oasis SPE cartridges were purchased from Waters (Milford, MA), and proteomics grade trypsin was from Promega (Madison WI). Trypsin-predigested β-galactosidase (a mass spectrometric quality control standard) was purchased from AB SCIEX (Foster City, CA).

### Bacterial strains and culture conditions

All bacterial strains used in this study are listed in **Table 4**. Derivatives were constructed by generalized transduction with P1kc, as described previously [79]. For strain construction, cells were grown in LB containing 1% (w/v) tryptone, 0.5% (w/v) yeast extract, and 0.5% (w/v) sodium chloride; LB plates also contained 1.5% agar. For Western immunoblot and mass spectrometric analyses, cells were grown at 37°C in TB7 [1% (w/v) tryptone buffered at pH 7.0 with potassium phosphate (100 mM)] or TB7 supplemented with 0.4% glucose or 25 mM acetate. Cell growth was monitored spectrophotometrically (DU640; Beckman Instruments, Fullerton, CA) by determining the absorbance at 600 nm (A_600_). Spectinomycin (100 µg/ml), ampicillin (100 µg/ml), and chloramphenicol (25 µg/ml) were added to growth media when needed.

**Table 4:** Bacterial strains used in this study.

Strain	Genetic Background	Relevant Characteristic	Source/Reference
MG1655	MG1655	*λ- rph-1*	A. Ninfa (University of Michigan)
AJW2012	MG1655	MG1655 Δ*ackA*::*kan*	P1: AJW1939 → MG1655
AJW2785	MG1655	MG1655 Δ(*ackA pta*)::*cat*	P1: EPB6 → MG1655
AJW3699	MG1655	MG1655 Δ*pta*::*FRT-kan-FRT*	P1: JW2294 → MG1655
AJW5035	MG1655	MG1655 Δ*yfiQ*::*FRT-kan-FRT*	P1: JW2568 →MG1655
AJW5037	MG1655	MG1655 Δ*cobB*::*cat*	P1: JE8659 → MG1655
AJW5052	MG1655	MG1655Δ*ackA*::*FRT*	P1: JW2293 → MG1655, cassette was removed
AJW5161	MG1655	MG1655 Δ*ackA*::*FRT* Δ*yfiQ*::*FRT-kan-FRT*	P1: JW2568 →AJW5052
AJW5164	MG1655	MG1655 Δ*yfiQ*::*FRT*	P1: JW2568 →MG1655, cassette was removed
AJW678	AJW678	*thi-1 thr-1* (Am) *leuB6 metF159*(Am) *rpsL136* Δ*lacX74*	[100]
AJW1939	AJW678	AJW678 Δ*ackA*:: *kan*	[100]
AJW2013	AJW678	AJW678 Δ(*ackA pta hisJ hisP dhu*) *zej223-*Tn*10*	[101]
BW25113	BW25113	*λ-* F^-^ *rph-1* Δ(*araD-araB)567 lacZ4787*Δ::*rrnB*-3 Δ(*rhaD-rhaB*)*568 hsdR514*	[102]
AJW2921	BW25113	BW25113 Δ*ackA*::*FRT-kan-FRT*	P1: JW2293 → BW25113
AJW2922	BW25113	BW25113 Δ*pta*::*FRT-kan-FRT*	P1: JW2294 → BW25113
PAD282	MC4100	F- *araD139* Δ(*argF-lac*)*U169 rpsL150*(*strR*) *relA1 flhD5301 deoC1* λΦ(*PcpxP*'*-lacZ*)	[103]
AJW2791	MC4100	PAD282 Δ(*ackA pta*)::*cat*	P1: EPB6 → PAD282
EPB6		Δ(*ackA pta*)::*cat*	Mark Goulian (University of Pennsylvania)
JE8659		Δ*cobB*::*cat*	Jorge Escalante-Semerena (U. of Georgia)
JW2293		Δ*ackA*::*FRT-kan-FRT*	[102]
JW2294		Δ*pta*::*FRT-kan-FRT*	[102]
JW2568		Δ*yfiQ*::*FRT-kan-FRT*	[102]

### Western immunoblot analysis of cell lysates

Overnight cultures were diluted into fresh TB7 at an OD600 of 0.1. Cells were harvested at OD600s of 0.5 and 1.0 and then at hours 8, 24 and 32. Cells were pelleted and lysed with 4X sample buffer (9% SDS, 0.36 M Tris pH 6.8, 45% glycerol, 2% bromophenol blue, 10% β-mercaptoethanol) and heated at 100°C for 10 minutes. Protein loading was normalized after protein amount was determined by BCA assay (Pierce 23225). Purified proteins were separated by SDS-PAGE with 4.6 M urea. The gel and the nitrocellulose membrane were rinsed in water and then equilibrated for 15 minutes in transfer buffer containing 25 mM Tris, 0.192 M glycine, and 20% (v/v) methanol. Proteins were transferred onto the membrane for 1 hour at 100 V. The blot was blocked with 5% (w/v) milk prepared in TBST for 2 hours at room temperature. The blot was washed with PBST two times for 5 minutes each. A rabbit polyclonal antibody raised against an acetylated lysine-containing peptide (Cell Signaling 9441) was used at a 1∶500 dilution in 5% BSA in PBST at 4°C overnight. The blot was washed 3 times for 5 minutes each and then one time for 10 minutes with PBST and then incubated with HRP-conjugated goat anti-rabbit secondary antibody (Cell Signaling 7074S) at a 1∶2000 dilution in 5% milk in TBST for 1 hour at room temperature. The blot was washed 4 times for 5 minutes each with PBST and exposed using 20X LumiGLO^R^ Reagent and Peroxide (Cell Signaling 7003).

### Cell lysis and proteolytic digestion of protein lysates

Cell pellets from WT and isogenic mutant strains grown in TB7 or TB7 supplemented with 4% glucose were suspended in 6 mL of PBS and centrifuged at 4°C, 15,000 g for 20 min. The firm cell pellet was collected and re-suspended and denatured in a final solution of 6 M urea, 100 mM Tris, 75 mM NaCl containing the deacetylase inhibitors 1 mM tricostatin A and 3 mM nicotinamide. Samples were sonicated on ice (5X each), cellular debris removed, and the supernatant for each sample further processed for proteolytic digestion. Typically 1.5 mg of protein lysate was reduced with 20 mM DTT (37°C for 1 hour), and subsequently alkylated with 40 mM iodoacetamide (30 min at RT in the dark). Samples were diluted 10-fold with 100 mM Tris pH 8.0 and incubated overnight at 37°C with sequencing grade trypsin (Promega) added at a 1∶50 enzyme:substrate ratio (wt/wt). Samples were then acidified with formic acid and desalted using HLB Oasis SPE cartridges (Waters) [80]. Proteolytic peptides were eluted, concentrated to near dryness by vacuum centrifugation, and re-suspended in NET buffer (50 mM Tris-HCl, pH 8.0, 100 mM NaCl, 1 mM EDTA). A small aliquot of each protein digestion (∼10 µg) was further desalted (C-18 zip-tips) for total protein lysate mass spectrometric analysis. The remaining tryptic peptide samples were used for affinity purification of lysine-acetylated peptides.

### Affinity purification of lysine-acetylated peptides

The polyclonal anti-acetyllysine agarose antibody conjugate from ImmuneChem (ICP0380-100) was prewashed and incubated with 1 mg *E. coli* digested protein lysate (in NET buffer) overnight (4°C) at a 1∶25 antibody:peptide ratio (wt/wt). Beads were washed three times in NET buffer, and the enriched acetyl peptides eluted by washing three times in 1% trifluoroacetic acid/40% acetonitrile. Samples were concentrated to near dryness by vacuum centrifugation and resuspended in equal amounts of 0.1% formic acid/1% acetonitrile. Prior to mass spectrometric analysis the acetyllysine peptide enrichment samples were desalted using C-18 Ziptips (Millipore, Billerica, MA).

### Mass spectrometric and chromatographic methods and instrumentation

Samples were analyzed by reverse-phase HPLC-ESI-MS/MS using the Eksigent Ultra Plus nano-LC 2D HPLC system (Dublin, CA) connected to a quadrupole time-of-flight TripleTOF 5600 mass spectrometer (AB SCIEX). Typically, mass resolution for MS1 scans and corresponding precursor ions was ∼35,000, while resolution for MS2 scans and resulting fragment ions was set at 15,000, a ‘high sensitivity’ product ion scan mode. Details for the mass spectrometric and chromatographic methods are provided in the Methods S1. Briefly, samples were acquired in data-dependent mode (DDA) on the TripleTOF 5600 to obtain MS/MS spectra for the 30 most abundant precursor ions following each survey MS1 scan. Single colonies were grown overnight for ∼15 hours in TB7 at 37°C with aeration, were diluted to an OD600 of 0.05 in 500 ml TB7 supplemented with or without 0.4% (w/v) glucose, and aerated for 7.5 hour in 1 L flasks at 37°C. The cells were pelleted at 4°C and washed with ice cold PBS, pelleted at 4°C again, and frozen in a dry ice and ethanol bath. The pellets were stored at −20°C before shipment on dry ice. For *E. coli* strains grown in TB7 containing 0.4% glucose, we analyzed three (*yfiQ*) or four (WT, *ackA, cobB*) independent biological replicates, each grown approximately 1 month apart. Each biological replicate was subjected to 3 technical MS replicates (i.e., three injection replicates on the mass spectrometer). Similarly, all strains grown without glucose supplement were analyzed in 3 independent biological replicates, each with 3 technical MS replicates.

For selected samples, additional data sets were recorded in data-independent mode (DIA) using MS/MS^ALL^ with SWATH acquisitions for quantitative analysis. During SWATH-MS2 acquisition, a wide window of ∼25 *m/z* is passed in Q1 incremental steps over the full mass range from *m/z* 400–1000 (see Methods S1). SWATH-MS2 analysis was performed to confirm MS1 Filtering quantitative results on one biological replicate (with three technical MS replicates) for WT and its isogenic *ackA* and *cobB* mutants, all grown in TB7 supplemented with 0.4% glucose.

### Bioinformatic database searches

Mass spectrometric data was searched using the database search engine ProteinPilot [81] (AB SCIEX Beta 4.5, revision 1656) with the Paragon algorithm (4.5.0.0, 1654). Mass spectral data sets also were analyzed using a Mascot [82] server version 2.3.02 after initially generating peak lists with the AB SCIEX mgf data converter version 1.3. All extensive details including search parameters, fixed and variable modifications, enzyme specificity, databases used, scoring, and false discovery rate analysis (FDR) are described in the Methods S1.

### Quantitative Skyline MS1 Filtering Analysis

MS1 chromatogram-based quantitation was performed in Skyline, an open source software project (http://proteome.gs.washington.edu/software/skyline) [83] using a MS1 ion intensity chromatogram processing called MS1 Filtering [38]. Briefly, prior to MS1 Filtering, comprehensive spectral libraries were first generated in Skyline from database searches of the raw data files using the BiblioSpec algorithm [84]. Second, all raw files acquired in data dependent acquisition mode (DDA) were directly imported into Skyline 1.4, and MS1 precursor ions were extracted for all peptides present in the MS/MS spectral libraries (the MS1 resolution setting in Skyline for precursor ion extraction is set at 10,000). Quantitative analysis was based on extracted ion chromatograms (XICs) and the resulting precursor ion peak areas for M, M+1, and M+2; final quantitative comparisons were typically based on only the highest ranked (naturally most abundant) precursor ion, which was then compared between samples.

### Quantitative SWATH Data Analysis in Skyline

Data sets from MS/MS^ALL^ with SWATH acquisitions were processed using the full scan MS/MS filtering module for data independent acquisitions within Skyline 1.4. Selected samples (1 biological replicate) containing acetylpeptides were subjected to SWATH-MS2 analysis (for confirmation of the quantitative MS1 Filtering results) and subsequently processed extracting the top 4-6 MS2 product ions using a product ion resolution setting of 10,000 (peak areas for the sum of the top 4-6 product ions are processed).

### Statistical Analysis of Quantitative MS data sets

All peak areas obtained from extracted precursor ion chromatograms using MS1 Filtering were exported from Skyline into excel sheets using the statistical program ‘MS1Probe’ [85], (http://www.gibsonproteomics.org/resources/MS1Probe). For each biological replicate and *E. coli* strain, the peak areas for individual acetyllysine peptides were automatically assembled in a data array to subsequently calculate peak area ratios per peptide between different strains, i.e. *ackA*/WT (for each strain, the 3 technical MS replicates were grouped). Significance was assessed using two-tailed Student's t-test requiring p-values<0.05. To assess possible regulation of acetylation in *E. coli* mutants relative to their WT parent, for a given acetyllysine-containing peptide, we set a threshold of at least a two-fold change in the relative peak area between the mutant and the WT with significance of p<0.05 within each biological replicate (each with 3 technical MS replicates). To be considered a ‘candidate’, such significant up-regulation was then required in at least 3 independent biological replicates out of the four biological replicates. Subsequently, for a given acetyllysine-containing peptide the mutant/WT ratio from each biological replicate was log-transformed, an average ratio over the 4 biological replicates with standard deviation and coefficient of variation (CV) calculated, and the obtained average log ratio was transformed back to a natural ratio for final reports. In cases where a specific acetyllysine site was identified with different precursor ion charge states or that produced multiple acetyllysine containing peptides with additional missed proteolytic cleavages or modifications, the most reproducible and most robustly ionizing species per site was chosen for quantification. To determine if the concentration of acetylated proteins that showed candidate acetylation sites for acetyl regulation was similar or different in the mutant relative to its WT parent, levels were assessed by quantifying non-modified peptides from the whole protein lysates.

### Data accession

The raw and processed data associated with this manuscript may be downloaded at ftp://sftp.buckinstitute.org:244/Ecoli_acetylome/. All details for peptide quantitation using MS1 Filtering, including peptide peak areas for all MS replicates, are provided as excel files.

A Skyline spectral library file that contains all MS/MS spectra of PTM-containing peptides identified and subsequently used for quantitation in MS1 Filtering, was transferred to the public data-sharing Panorama site [86]. Panorama provides an interactive web-based spectral viewer of all acetyllysine-containing peptides identified in this study (at 99% confidence and with expectation value <0.01, respectively). The spectral viewer can be accessed at http://proteome.gs.washington.edu/software/panorama/gibson_ecoli_acetylome.html. All MS/MS spectral details underlying this data also are displayed in **Tables S6 and S7**. Acetylation site assignment was initially suggested by search engines ProteinPilot and Mascot and confirmed by manual inspection using previously defined criteria [87].

### Functional Analysis, Protein Ontology, Pathway Enrichment analysis

For functional analysis and protein ontology analysis ‘The Database for Annotation, Visualization and Integrated Discovery’ (DAVID v.6.7) was used [41]. For pathway enrichment analysis, we used the Bonferroni correction as adjustment to p-values to determine statistical significance. To generate graphical ontology displays, Panther v8.1 classification system was used [40].

### Synthesis of Peptide Libraries

Individual peptides were synthesized in 96 well filter plates, using methods previously described [88]. A library was constructed in the format Ac-GXKZGC-CONH_2_, where X and Z positions represent all amino acids except cysteine. A total of 361 peptides were synthesized and stored in 0.1% TFA in water in a 384 well plate.

### Preparation of SAMDI Peptide Arrays

The Ac-GXKZGC-CONH_2_ peptide library was diluted into 50 mM Tris-HCl pH 8.0 to a final concentration of 20 µM in a 384 well format. A Tecan EVO 384 head was used to transfer 3 µl of buffered peptide to 384 SAMDI biochips of self-assembled monolayers on gold, prepared as previously described [89]. The cysteine-terminated peptides were captured onto monolayers presenting maleimide as a capture moiety. Peptides were immobilized onto surfaces for 1 h at room temperature. SAMDI Biochips were then washed with copious amounts of 100% ethanol, deionized ultra-filtered (DIUF) water, 100% ethanol, and then dried with nitrogen gas. Peptides were tested by SAMDI.

### AcP SAMDI Assays

For acP reactions, SAMDI biochip arrays with the immobilized Ac-GXKZGC-CONH_2_ peptide library were subjected to a mixture of 50 mM acP, 150 mM NaCl, 10 mM MgCl_2_ and 50 mM Tri-HCl pH 8.0. A total of 3 µl of the solution was transferred to the biochips and incubated for 1 h at 30°C. A negative control containing the same reaction buffer in the absence of AcP was used to determine the background level of acetylation. Reactions for AcP and controls were performed in duplicate and were quenched by washing biochips with 100% ethanol prior to SAMDI analysis.

### SAMDI Mass Spectrometry

A 3 ml solution of 2′,4′,6′-trihydroxyacetophenone monohydrate (THAP) matrix (30 mg/ml in acetone) was applied to the biochips and allowed to dry prior to SAMDI. Mass analysis was performed using a 5800 MALDI-TOF/TOF (AB Sciex). A 355 nm Nd:YAG laser was used as a desorption/ionization source, and all spectra were acquired with 20 kV accelerating voltage using positive reflector mode. The extraction delay was 700 ns, 500 laser shots were applied, and the entire surface of the spot was randomly sampled. Each acquired spectrum was examined for complete peptide immobilization by monitoring the conversion of the maleimide monolayer. The extent of the acP reactions was calculated as previously reported [48].

### *In vivo* consensus sequence analysis

A consensus sequence for acP-regulated lysine acetylation was generated using the mass spectrometric data. Acetylated lysines that were significantly up-regulated (ratio>2, p-value<0.05) in at least 3 out of 4 biological replicates of the *ackA* mutant relative to WT cells were used in this analysis. Of the 592 unique sites that fit this description, 28 and 24 sites were less than 10 residues from the N-terminus or the C-terminus; these were removed from the following analysis, leaving 541 acetylated sites. Sequences of 21 amino acids, representing 10 amino acids N-terminal and 10 amino acids C-terminal relative to the acetylated lysine were analyzed and a consensus logo was generated using WebLogo [50].

### Protein Expression, Purification, and Crystallization

ASKA clones of *gapA, tpiA* and *lpdA* from *E. coli* K-12 were used to produce protein for crystallization experiments. The clones contain a six-histidine N-terminal affinity tag followed by TDPALRA spacer residues and then the ORF of the gene ligated into the pCA24N expression vector as described [90]. Proteins were expressed in BL-21 (DE3) Magic cells and purified using previously described protocols [29], [91], [92].

An additional purification step was introduced for the TpiA protein. First, 30 mg of concentrated purified protein (10 mM Tris-HCl pH 8.3, 500 mM NaCl, 5 mM beta-mercaptoethanol) was diluted into 50 mM Tris-HCl pH 8.3 buffer to obtain a final salt concentration of 150 mM NaCl. Half of this sample was used as a control with no modification and the other half was used for a reaction with AcP. Two ml of protein sample were first incubated with 50 mM acP for 30 min at 30°C. The sample was then incubated a second time with 50 mM acP in a final volume of 3 ml for 30 min at 30°C. The samples were diluted to 50 ml in 50 mM Tris pH 8.3 and 150 mM NaCl and loaded onto an 8 ml MonoQ 10/10 GL column (GE Healthcare). Using a linear gradient from 0-70%, the proteins were eluted and fractions collected. Each peak of native TpiA and acP-treated TpiA was concentrated and used for crystallization trials with the Classics II and PACT screens (Qiagen). The PACT screen (Qiagen) was used for crystallization trials of GapA. All crystallizations were performed in a 96-well plate with 100 µl of reservoir solution and 1∶1 µl reservoir to protein (7.3 mg/ml for GapA and 7.4 mg/ml for TpiA) at 22°C. Crystals grew within a month.

### Data Collection and Structure Determination

Crystals were harvested from PACT D11 (0.2 M calcium chloride, 0.1 M Tris pH 8, 20% (w/v) PEG 6000) for native TpiA, Classics F11 (0.2 M NaCl, 0.1 M Bis-Tris pH 6.5, 25% (w/v) PEG 3350 for acP-treated TpiA, and PACT G2 0.2 M sodium bromide, 0.1 M Bis Tris propane 7.5, 20% (w/v) PEG 3350 for AcP-treated GapA. Crystals not treated with acP were transferred to 5 µl of reservoir solution and mounted and frozen in liquid nitrogen for data collection. The crystals from Classics F11 and PACT G2 were treated with acP by soaking using the following procedure: crystals were transferred to 5 µl of the reservoir solution, AcP powder was added directly to the solution (approximately 10 mM final concentration) using a pipette tip, and crystals were quickly drawn through the solution, mounted and flash frozen with liquid nitrogen. The time between adding the acP and plunging the crystal into liquid nitrogen was less than 30 sec. Data were collected at 100 K at the Life Sciences Collaborative Access Team (LS-CAT) 21ID-D, 21ID-F and 21ID-G beamlines at Argonne National Laboratory (Argonne, IL). HKL3000 [93] was used for data processing, integration, and scaling. The GapA and TpiA structures were solved by Molecular Replacement with Phaser [94] in the CCP4 suite [95], using the GapA structure from *E. coli* (PDB ID: 1S7C) and the TpiA structure from *E. coli* (PDB ID: 1TRE) as the search models. The models were refined using Refmac [96] and acetylated lysines were modified using the CCP4 monomer library. Manual corrections were performed on workstations using COOT [97] and the final models were validated using the validation server of the PDB. PDB Codes: 4K6A (native tpiA), 4MVA (acP-treated TpiA), and 4MVJ (acP-treated GapA). Figures were made using Pymol (Delano, 2002) and structure parameters are shown in **Table S29**.

### Structural Analysis of Proteins Modified by AcP in the ackA mutant

The following *E. coli* crystal structures were used for mapping lysines on glycolytic enzymes that were modified in the *ackA* mutant: PfkB (PDB ID: 3UQD) with ATP and fructose 6-phosphate (F6P); Fbp (PDB ID: 2OWZ) with F6P; GlpX (PDB ID: 3DIR) with fructose 1,6-bisphosphate (FBP); FbaA (PDB ID: 1B57) with phosphoglycolohydroxamic acid (PGH); TpiA (PDB ID: 4K6A, 4MVA) with acP; GapA (PDB ID: 1DC4 and 1DC6) with G3P from 1DC4 and NAD^+^ from 1DC6; Pgk (PDB ID: 1ZMR) with phosphoaminophosphonic acid-adenylate ester (ANP) and 3-phosphoglyceric acid (3PG) of 1VPE; GpmA (PDB ID: 1E59) with VO_3_; Eno (PDB ID: 1E9I) with SO_4_; and PykF (PDB ID: 1PKY) without a ligand.

### *In vitro* acetylation with acP

1.25 µM of LpdA and various concentrations of acP were incubated at 37°C in 150 mM Tris HCl (pH 7.3 at room temperature), 10% glycerol, 10 mM MgCl_2_, and 150 mM NaCl at the indicated amount of time. For each acP concentration, time points were prepared at 0, 5, 10, 15, 30 min and at 1, 2, 3, 4, 5, 7 hours. To assess variability, each of the 4 time courses (at the 4 acP concentrations) was performed in ‘incubation duplicates.’ To stop the reaction, an equal volume of 2X SDS loading buffer was added and the reactions were heated at 95°C for 10 minutes.

RNAP was purified as described [30]. 1.25 µM of RNAP complex were incubated with acP at 10 mM in the acP *in vitro* acetylation buffer described for LpdA and acP. Time points at 0 min and at 30 min were collected. To assess variability, the experiments were performed in ‘incubation duplicates.’

### Western immunoblot analysis of purified proteins

Following *incubation* with acP, purified proteins were separated by SDS-PAGE. The gel and the nitrocellulose membrane were equilibrated for 15 minutes in transfer buffer containing 25 mM Tris, 0.192 M glycine, 20% (v/v) methanol, and 0.1% (w/v) SDS. Proteins were transferred onto the membrane for 30 min using a Biorad TurboTransfer. The blot was blocked with 5% (w/v) milk prepared in TBST for 1 hour at room temperature. The blot was washed with TBST for 5 min. An anti-acetylated lysine-containing peptide (Cell Signaling 9441) was used at a 1∶1000 dilution in 5% BSA in TBST at 4°C overnight. The blot was washed 3 times for 5 min each with TBST and then incubated with HRP-conjugated goat anti-rabbit secondary antibody (Cell Signaling 7074S) at a 1∶1000 dilution in 5% milk in TBST for 1 hour at room temperature. The blot was washed 3 times for 5 min each with TBST and exposed using 20X LumiGLO^R^ Reagent and Peroxide (Cell Signaling 7003). Quantification of acetylation in terms of average pixel density was performed using AlphaView.

### Electrophoretic Separation and Mass Spectrometric Analysis of *in vitro* acetylated proteins

Samples (from different acP concentrations and different time points) were electrophoresed, each separated by an empty lane(s) to avoid cross contamination as previously described [29]. To limit cross-contamination between samples, each gel lane was cut out and stained in its own container with SimplyBlue™ SafeStain (Invitrogen) and subsequently destained with water, or gels were stained with Coomassie Brilliant Blue and destained using methanol and acetic acid. The bands containing LpdA were excised. The RNAP complex was separated by electrophoresis and 4 distinct bands (RpoB/RpoC, RpoD, RpoA, and RpoZ) were excised. Subsequently, all gel bands were subjected to tryptic in-gel digestion as previously described [98]. Mass spectrometric analysis was performed on the TripleTOF 5600 in data-dependent mode with a total acquisitions runtime of 90 min per sample. For LpdA incubations, we analyzed the following: Series A - 10 mM acP at time points 0, 10, 60, 240, 420 min. Series B - 20 mM acP at time points 0, 10, 60, 240, 420 min. For each series, two independent incubation replicates were analyzed as two technical injection replicates. For RNAP incubations (10 mM acP), we analyzed time points at 0 and 30 min in two independent incubation replicates and two technical injection replicates.

### pKa Calculations and surface accessibility

pKa values for LpdA and RNAP subunits were calculated using PROPKA 3.1 at http://propka.ki.ku.dk/ [99]. PROPKA also calculates surface accessibility, defining a parameter referred to as buried ratio. As input for PROPKA, the following PDB structural files were used, PDB ID: 4JDR for LpdA, PDB ID: 4IGC for RNAP chain A (RpoA), chain C (RpoB), chain D (RpoC), and chain X (RpoD).

## Supporting Information

Figure S1 **Anti-acetyllysine Western immunoblot analyses.** **A**) *E. coli* WT (strain BW25113) and isogenic mutants *ackA* (strain AJW2921) and *pta* (strain AJW2922), each aerated at 37°C in TB7 and harvested at 5 time points, when the OD_610_ reached 0.5 or 1.0, and then at 8, 24 and 32 hours. **B**) *E. coli* WT (strain MG1655) and isogenic mutants *ackA* (strain AJW5052), *ackA yfiQ* (strain AJW5161), and *yfiQ* (strain AJW5035), each aerated at 37°C in TB7 and harvested at 3 time points, when the OD_610_ reached 0.5 or 1.0, and then at 8 hours. **C**) Long exposure of *E. coli* WT (strain PAD282) and isogenic mutant *pta ackA* (strain AJW2791), each aerated at 37°C in TB7 and harvested at 5 time points, when the OD_610_ reached 0.5 or 1.0, and then at 8, 24 and 32 hours.
(TIF)

Figure S2 **Distribution of lysine acetyl sites in** ***E. coli*** **proteins.** Mass spectrometric analysis of WT and various mutant strains confidently identified 2730 unique lysine acetylation sites across 806 unique acetylated *E. coli* proteins. **A**) The number of identified acetyl sites per protein compared to the protein molecular weight showed no correlation. The linear regression trendline is indicated (R^2^ = 0.08). **B**) The number of identified acetyl sites per protein compared to the number of all lysine residues within a protein showed a slight correlation. The linear regression trendline is indicated (R^2^ = 0.21). **C**) Number of proteins relative to percentage of lysines acetylated (acetyl site count/total lysine count).
(TIF)

Figure S3 **Label-free mass spectrometric quantification to assess lysine acetyl sites in** ***E. coli*** **.** **A**) Skyline MS1 Filtering (Schilling, B. et al., Mol Cell Proteomics: 11, 202-214 (**2012**)) to quantify MS1 signal intensities of peptide precursor ions across the chromatographic gradient for the entire *m/z* range. For each peptide, an isotopic envelope for the molecular ion with peaks at M, M+1, and M+2 was selected showing changes in MS1 intensity over time. Extracted ion chromatograms for molecular ion isotopes were generated and the area under the curve across the peptide elution profile was integrated. An example for one (of four) biological replicate (here BioRep 2) is shown for acetylated peptide TACEVAEISY**Kac**K with ^Ac^K101 from cAMP receptor protein (CRP) (precursor ion at *m/z* 720.86) for the WT and *ackA* mutant strains, each sample acquired in technical triplicates. Ion intensity for precursor ions for AckA MS replicates 1-3 were increased compared to WT. The integrated peak areas from BioRep 2 were then used to assess changes for acetylated lysine ^Ac^K101 and plotted for both the WT and *ackA* mutant strains; peak area means were determined to be 15,672 (WT) and 71,699 (*ackA*), revealing a 4.6 fold increase in ^Ac^K101 with a significant p-value of 9.32e^−6^. Biological replicates 1, 3 and 4 were processed similarly. **B**) To assess statistically significant regulation of acetylation in *E. coli* mutants relative to their WT parent, a threshold of ≥2-fold change with a p-value<0.05 was used within each biological replicate (with 3 technical MS replicates each). To be considered a ‘candidate’, significant up-regulation was required in at least 3 of the 4 independent biological replicates. To calculate mean ratios (mutant/WT) across biological replicates individual ratios were log transformed, averaged, and the resulting mean was finally transformed back into natural numbers and a coefficient of correlation (CV) was determined. Several examples are shown. Complete lists of quantifications/regulated targets are supplied in Tables S4A and S4B. *, Site was identified by MS but could not be quantified in one (Bio3) of four biological replicates.
(TIF)

Figure S4 **Comparison between** ***ackA*** **- and** ***cobB*** **-sensitive lysine acetyl sites.** Of the 2730 unique acetylation sites from 806 proteins identified from acetyllysine-enriched fractions, quantitative mass spectrometry could be performed for 2367 quantifiable sites for the *ackA* mutant, and 1392 quantifiable sites for the *cobB* mutant. For each quantifiable site, the acetyl site ratio (mutant/WT) was calculated and the frequency of fold change for all quantifiable acetyl sites was plotted: **A**) *cobB*/WT and **B**) *ackA*/WT. For the *ackA* mutant (**B**), the fold-change profile for up-regulated acetyllysines was broader and more global than that of the *cobB* mutant (**A**), which exhibited profile changes for a smaller number of distinct sites (see **Tables S15 and S16**). Subsequently, further stringent statistical requirements were applied to identify statistically significant and robustly regulated acetyl sites (see **Table S9**). **C**) The acetyl site ratio (*cobB*/WT) for acetylated lysines in proteins Crr, CsrA, GadA, GreA, YifE, and YihD (red, >2 fold and statistically significant; blue, <2 fold). **D**) The acetyl site ratio (*ackA*/WT) for acetylated lysines in proteins GatY, RaiA, RplL, RpsC, DnaK, and LpdA (red, >2 fold and statistically significant; brown, >2 fold but not statistically significant; blue, <2 fold). **E**) Direct comparison of acetylated lysines from three selected proteins in the *cobB* mutant and the *ackA* mutant each relative to WT. For the proteins GadA, HupB and InfA, the acetyl site ratios (*cobB*/WT and *ackA*/WT) are presented (red, >2 fold and statistically significant; brown, >2 fold but not statistically significant; blue, <2 fold). For panels C, D, and E, the black broken line represents an acetyllysine mutant/WT peak area ratio of 1 (i.e., no change).
(TIF)

Figure S5 **Validation by SWATH-MS2 analysis of quantification by MS1 Filtering.** Acetylated lysines determined by Skyline MS1 Filtering to be robustly up-regulated in the *ackA* relative to WT (592 acetyl sites) were subjected to SWATH-MS2 data-independent assays for validation. **A**) Global comparison between MS1 Filtering (MS1 quantification) and SWATH (MS2 quantification) determining fold changes (*ackA*/WT) for 526 acetyllysine sites that were determined to be significant by MS1 Filtering and also could be monitored independently by SWATH acquisitions. The data points showed the following distributions for the fold change (*ackA*/WT) using the two different quantification approaches: median for MS1 = 3.8 and MS2 = 4.3; the 25 percentile was at 3.1 for MS1 and 3.3 for MS2, while the 75 percentile was at 4.8 for MS1 and 5.7 for MS2. **B**) Comparison of quantification by MS1 Filtering (red squares) and by SWATH-MS2 (green circles) for acetyl site ratios (*ackA*/WT) from proteins LpdA, RcsB, RpoB, and RpoC that confirm many significant acetyl site changes. For both panels (**A and B**), the MS1 quantification was performed on 3 technical replicates of 4 biological replicates, while the MS2 quantification was performed on 3 technical replicates of 1 biological replicate. The blue broken line represents an acetyllysine mutant/WT peak area ratio of 1 (i.e., no change).
(TIF)

Figure S6 **MS1 quantification of acP-dependent acetylation.** **A**) Pta-AckA pathway schematic. **B**) Anti-acetyllysine antibody Western immunoblot analysis of lysates obtained from the mutants *ackA* (strain AJW2012) and *pta ackA* (strain AJW2785) grown in the absence of glucose. **C**) Quantitative MS1 Filtering peak area view of a selected acetylated peptide (GQVLA**Kac**PGTIKPHTK with ^Ac^K295 from protein EF-Tu) for one selected biological replicate (out of three) with 3 technical MS replicates, comparing relative abundance of the acetylated peptide in the *ackA* strain relative to its abundance in the *pta ackA* strain (*ackA*/*pta ackA*), each grown without glucose. **D**) Distribution analysis of statistically significant fold changes determined for 84 acetyl sites across 3 biological replicates grown in the absence of glucose.
(TIF)

Figure S7 **Ontology assessment of** ***ackA*** **-sensitive lysine acetyl sites.** 292 proteins that exhibited significant up-regulation for their 592 acetyl sites in the *ackA* mutant relative to WT were subjected to ontology analysis using Panther. **A**) Molecular function GO categories, and **B**) Biological processes GO categories.
(TIF)

Figure S8 **Central metabolic enzymes with** ***ackA*** **-sensitive lysine acetyl sites.** 292 proteins that exhibited significant up-regulation for their 592 acetyl sites in the *ackA* mutant relative to WT were subjected to DAVID ontology and pathway enrichment analysis. Acetylated proteins that were significantly enriched for central metabolic pathways are indicated with green asterisks in the schematic (adapted from J.L. Báez-Viveros et al, *Microbial Cell Factories*, **2007**).
(TIF)

Figure S9 **Monitoring acetylated lysine residues due to incubation of recombinant LpdA with 10 mM or 20 mM acP.** **A**) 1.25 µM purified LpdA was incubated with either 10 mM or 20 mM acP over a defined time course. At time points 0, 5, 10, 15, and 30 min and at 1, 2, 3, 4, 5, and 7 hours, samples were subjected to quantitative MS1 Filtering analysis and relative abundance changes for each LpdA acetylation site monitored. All individual ratios were related to the zero time point (n/0 min) and were calculated from two incubation replicates (B1 and B2), each acquired in technical MS duplicates. **B**) Quantitative results for specific LpdA sites ^Ac^K278 (**i and iii**) and ^Ac^K284 (**ii and iv**) during incubation with 10 mM acP (**i and ii**) or 20 mM acP (**iii and iv**). For each time point, ratios (n/0 min) are shown for both incubation replicates B1 (**red**) and B2 (**blue**), each acquired in technical MS duplicates. Peptides quantified using MS1 Filtering were i) VPNG**Kac**NLDAGK (^Ac^K278) at 10 mM acP, ii) NLDAG**Kac**AGVEVDDR (^Ac^K284) at 10 mM acP, iii) VPNG**Kac**NLDAGK (^Ac^K278) at 20 mM acP, iv) NLDAG**Kac**AGVEVDDR (^Ac^K284) at 20 mM acP.
(TIF)

Figure S10 **Monitoring acetylated lysine residues from incubation of purified RNAP with 10 mM acP.** 1.25 µM purified RNAP was incubated with 10 mM acP for 30 min. MS1 Filtering was used to quantify relative changes over time in the abundance of acetyl sites; ratios for acetyl sites (30 min/0 min) were calculated from two incubation replicates (B1 and B2), each acquired in technical MS duplicates. Results are presented for K297 of the α subunit (RpoA, monitoring acetyl peptide TPNLG**Kac**K), K1065 of the β subunit (RpoB, monitoring acetyl peptide IQPGD**Kac**MAGR), and both K493 and K593 of the σ^70^ subunit (RpoD, monitoring acetyl peptides MLMPED**Kac**IR and QIEA**Kac**ALR, respectively). The following fold changes in relative acetylation abundance across both incubation and MS technical replicates were observed: 4.83-fold for ^Ac^K297 (0.5% CV); 14.84-fold for ^Ac^K1065 (4% CV); 4.71-fold for ^Ac^K493 (17% CV); and 6.71 for ^Ac^K593 (11% CV).
(TIF)

Table S1 **Overview of all 2730 unique** ***E. coli*** **acetyllysine sites identified from all strains and under all growth conditions.**
(XLSX)

Table S2 **Overview of unique acetyllysine sites for specific strains and growth conditions: WT grown with glucose.**
(XLSX)

Table S3 **Overview of unique acetyllysine sites for specific strains and growth conditions:** ***ackA*** **mutant grown with glucose.**
(XLSX)

Table S4 **Overview of unique acetyllysine sites for specific strains and growth conditions:** ***cobB*** **mutant grown with glucose.**
(XLSX)

Table S5 **Overview of unique acetyllysine sites for specific strains and growth conditions: all corresponding acetylated proteins containing lysine acetyl sites for all strains combined (and individual strains/conditions).**
(XLSX)

Table S6 **Mass spectrometric details for all identified acetyllysine-containing peptides as identified using the AB SCIEX Protein Pilot search engine.**
(XLSX)

Table S7 **Mass spectrometric details for all identified acetyllysine-containing peptides as identified using the Mascot v.2.3 search engine (Matrix Sciences).**
(XLSX)

Table S8 **Acetylation site distribution per protein: frequency of acetyl sites observed within individual proteins.** The number of lysine acetyl sites per protein relative to the estimated protein abundance (copy number per cell), to protein molecular weight, and to the total number of lysines per protein.
(XLSX)

Table S9 **Quantitative MS1 Filtering identified statistically significant increased acetylation of lysines sites in mutants relative to WT: Significant upregulation of 592 unique ackA acetylation sites.**
(XLSX)

Table S10 **Quantitative MS1 Filtering identified statistically significant increased acetylation of lysines sites in mutants relative to WT: significant upregulation of 69 unique cobB acetylation sites.**
(XLSX)

Table S11 **Quantitative MS1 Filtering identified statistically significant increased acetylation of lysines sites in mutants relative to WT: List of 292 proteins that correspond to the 592 sites that were significantly regulated in the** ***ackA*** **mutant relative to WT.**
(XLSX)

Table S12 **Quantitative MS1 Filtering identified statistically significant increased acetylation of lysines sites in mutants relative to WT: list of 51 proteins that correspond to the 69 sites that were significantly regulated in the** ***cobB*** **mutant relative to WT.**
(XLSX)

Table S13 **Quantitative MS1 Filtering of acetylated proteins on the protein lysate level (quantification of non-modified peptides) for proteins that contained acetyl sites upregulated in the** ***ackA*** **mutant relative to WT.**
(XLSX)

Table S14 **Quantitative MS1 Filtering of acetylated proteins on the protein lysate level (quantification of non-modified peptides) for proteins that contained acetyl sites upregulated in the** ***cobB*** **mutant relative to WT.**
(XLSX)

Table S15 **MS1 Filtering for all lysine acetyl sites in mutant strains relative to WT, including acetyl sites that change and those that do not:** ***ackA*** **- 2367 quantifiable acetyl sites.**
(XLS)

Table S16 **MS1 Filtering for all lysine acetyl sites in mutant strains relative to WT, including acetyl sites that change and those that do not:** ***cobB*** **- 1392 quantifiable acetyl sites.**
(XLS)

Table S17 **Independent verification of MS1 Filtering data using SWATH-MS2.** Data independent acquisition and quantitation using MS2 fragments in a SWATH method as used to independently verify 592 candidate *ackA*-sensitive acetyl sites.
(XLSX)

Table S18 **Independent verification of MS1 Filtering data using SWATH-MS2.** Data independent acquisition and quantitation using MS2 fragments in a SWATH method as used to independently verify 69 candidate *cobB*-sensitive acetyl sites.
(XLSX)

Table S19 **Independent verification of MS1 Filtering data using SWATH-MS2.** The complete list of MS2 fragment ions used for Table S17.
(XLSX)

Table S20 **Independent verification of MS1 Filtering data using SWATH-MS2.** The complete list of MS2 fragment ions used for Table S18.
(XLSX)

Table S21 **Quantitative MS1 Filtering of acP-dependent acetylation, identifying acetyl sties that were increased significantly in the** ***ackA*** **mutant relative to the isogenic** ***pta ackA*** **mutant, both grown in the absence of glucose.** Three biological replicates were acquired in three technical MS replicates.
(XLSX)

Table S22 **Ontology Assessment of 292 acetyllysines upregulated in the** ***ackA*** **mutant relative to its WT parent using Panther: molecular function GO categories.**
(XLSX)

Table S23 **Ontology Assessment of 292 acetyllysines upregulated in the** ***ackA*** **mutant relative to its WT parent using Panther: biological processes GO categories.**
(XLSX)

Table S24 **Pathway enrichment analysis of 292 acetyllysines upregulated in the** ***ackA*** **mutant relative to its WT parent, as determined by DAVID Ontology: Gene Ontology [GOTERM_BP_FAT (DAVID)] analysis.**
(XLSX)

Table S25 **Average and standard deviation of acP-dependent peptide acetylation by SAMDI assay data after background subtraction.**
(XLSX)

Table S26 **Frequency table for** ***in vivo*** **sequence logo.** Amino acid frequencies relative to the acetylated lysine were analyzed. 541 sites that exhibited significantly upregulated sites in the ackA mutant relative to WT with a p-value<0.05 were analyzed. Acetylated lysines that were within 10 amino acids of the start (28 sites) or stop codon (24 sites) were not part of this analysis.
(XLSX)

Table S27 **Normalized frequency table for** ***in vivo*** **sequence logos generated for lysines from LpdA, PflB, AceE, and GroL.** Proteins that possessed at least 10 lysines that were significantly acetylated in the *ackA* mutant relative to WT (P value<0.05) (LpdA, PflB, AceE, and GroL) were analyzed. Amino acid frequencies adjacent to lysines that were *ackA*-sensitive and *ackA*-insensitive were determined. These values were normalized by calculating the fold difference relative to the total frequency for each individual amino acid in LpdA, PflB, AceE, and GroL (freq(amino acids)).
(XLSX)

Table S28 **Relative prevalence of residues adjacent to either acP-upregulated or non-acP-regulated lysines.** **A–F** compare the relative frequencies for residues in the −1 and +1 positions at *ackA*-sensitive and *ackA*-insensitive LpdA, PflB, AceE, and GroL sites. We assessed statistical significance using two-tailed Student's t-Test and the p-values are shown. **A**) A comparison of the frequencies of top three most frequent residues in the -1 position at *ackA*-sensitive sites (C, E, and D) to the frequencies of these residues at *ackA*-insensitive sites. **B**) A comparison of the top three most frequent residues in the +1 position at *ackA*-sensitive sites (L, D, and N) to the frequencies of these residues at *ackA*-insensitive sites. **C**) A comparison of the top three most frequent residues in the -1 position at *ackA*-insensitive sites (K, S, and P) to the frequencies of these residues at *ackA*-sensitive sites. **D**) A comparison of the top three most frequent residues at *ackA*-insensitive sites (V, K, and F) to the frequencies of these residues at *ackA*-sensitive sites. **E**) A comparison of the negative (E and D) and positive (K and R) residues in the -1 positions at *ackA*-sensitive and -insensitive sites. **F**) A comparison of the negative (E and D) and positive (K and R) residues in the +1 positions at ackA-sensitive and -insensitive sites.
(XLSX)

Table S29 **Crystallography data collection and refinement statistics.**
(DOCX)

Table S30 **MS1 Filtering and quantification of acetylysine sites following** ***in vitro*** **incubation of purified recombinant LpdA with 10 mM acP**.
(XLSX)

Table S31 **MS1 Filtering and quantification of acetylysine sites following** ***in vitro*** **incubation of purified recombinant LpdA with 20 mM acP.**
(XLSX)

Table S32 **MS1 Filtering and quantification of acetylysine sites following** ***in vitro*** **incubation of purified RNAP with 10 mM acP.**
(XLSX)

Table S33 **Calculated pKa values and surface exposure (buried ratio) of LpdA lysines that are acetylated** ***in vitro*** **by acP.**
(XLSX)

Table S34 **Calculated pKa values and surface exposure for all LpdA Lysines.**
(XLSX)

Table S35 **Calculated pKa values and surface exposure (buried ratio) for lysines of RNAP subunits RpoA (α), RpoB (β), and RpoD (σ^70^) that become acetylated when incubated with acP** ***in vitro*** **.**
(XLSX)

Table S36 **Calculated pKa values and surface exposure for all lysines of subunits RpoA (α), RpoB (β), RpoC (β′) and RpoD (σ^70^).**
(XLSX)

Methods S1 **Mass spectrometric and chromatographic methods.** Samples were analyzed by reverse-phase HPLC-ESI-MS/MS using an Eksigent Ultra Plus nano-LC 2D HPLC system (Dublin, CA), which was directly connected to a quadrupole time-of-flight (QqTOF) TripleTOF 5600 mass spectrometer (AB SCIEX, Concord, CAN) in direct injection mode. The autosampler was operated in full injection mode overfilling a 1 µl loop with 3 µl analyte for optimal sample delivery reproducibility. Briefly, after injection, peptide mixtures were transferred onto the analytical C18-nanocapillary HPLC column (C18 Acclaim PepMap100, 75 µm I.D. ×15 cm, 3 µm particle size, 100 Å pore size, Dionex, Sunnyvale, CA) and eluted at a flow rate of 300 nL/min using the following gradient: at 5% solvent B in A (from 0-13 min), 5–35% solvent B in A (from 13–58 min), 35–80% solvent B in A (from 58–63 min), at 80% solvent B in A (from 63–66 min), with a total runtime of 90 min including mobile phase equilibration. Solvents were prepared as follows, mobile phase A: 2% acetonitrile/98% of 0.1% formic acid (v/v) in water, and mobile phase B: 98% acetonitrile/2% of 0.1% formic acid (v/v) in water. Data-dependent acquisitions (DDA): Mass spectra and tandem mass spectra were recorded in positive-ion and “high-sensitivity” mode with a resolution of ∼35,000 full-width half-maximum on the TripleTOF 5600. The nanospray needle voltage was typically 2,500 V in HPLC-MS mode. After acquisition of ∼5 to 6 samples, the MS and MS/MS spectra were calibrated during dynamic LC-MS & MS/MS autocalibration acquisitions injecting 25 fmol beta-galactosidase. For collision induced dissociation tandem mass spectrometry, the mass window for precursor ion selection of the quadrupole mass analyzer was set to ±1 *m/z*. The precursor ions were fragmented in a collision cell using nitrogen as the collision gas. In DDA mode, the 30 most abundant parent ions were selected for MS/MS analysis following each MS1 survey scan (approx. 50 msec per MS/MS). Dynamic exclusion features were based on value M not *m/z* and were set to an exclusion mass width 50 mDa and an exclusion duration of 30 sec. Data-independent acquisitions (DIA), MS/MS^ALL^ with SWATH acquisitions: In the SWATH-MS2 acquisition, a Q1 window of 25 *m/z* was selected to cover the mass range of m/z 400–1000 in 24 segments (24×100 msec), yielding a cycle time of 3.25 sec, which includes one 250 msec MS1 scan. SWATH-MS2 produces complex MS/MS spectra, which are a composite of all the analytes within each selected Q1 *m/z* window. ***Bioinformatic database searches*** Mass spectrometric data was analyzed using ProteinPilot (Shilov, I.V. et al. Mol. Cell. Proteomics: 6, 1638–1655, **2007**) (AB SCIEX Beta 4.5, revision 1656) and the Paragon algorithm (4.5.0.0,1654). The following sample parameters were used: trypsin digestion, cysteine alkylation set to iodoacetamide, urea denaturation, acetylation emphasis, and species *E. coli*. Trypsin specificity was set at C-terminal lysine and arginine. Processing parameters were set to "Biological modification" and a thorough ID search effort was used. During the search, ProteinPilot performs an automatic mass recalibration of the data sets based on highly confident peptide spectra. Specifically, a first search iteration is done to select high confidence peptide identifications that are used to recalibrate both the MS and MS/MS data, which is automatically re-searched. All data files were searched using the SwissProt 2012_07 database) with a total of 8870 *E. coli* protein sequences. For ProteinPilot Searches, to assess and restrict rates of false positive peptide/protein identifications, we used the Proteomics System Performance Evaluation Pipeline (PSPEP) tool available in ProteinPilot 4.5 beta. This tool automatically creates a concatenated forward and reverse decoy database, and provides an Excel file output of the experimentally determined false discovery rate at the spectral, peptide and protein levels with standard statistical errors. The discriminating variable for the Paragon Algorithm is the peptide confidence value, which is a 0–99 scaled real number (Shilov, I.V. et al. Mol. Cell. Proteomics: 6, 1638–1655, **2007**). For database searches, a cut-off peptide confidence value of 99 was chosen with the following justification. For searching the databases, the Protein Pilot false discovery rate (FDR) analysis tool (PSPEP) algorithm (Shilov, I. V. et al. Mol. Cell. Proteomics: 6, 1638–1655, **2007**) provided a global FDR of 1% and a local FDR at 1% in all cases. Mass spectral data sets also were analyzed and searched using Mascot. In Mascot (Perkins, D.N. et al., Electrophoresis 20, 3551–3567, **1999**), peak lists were generated using the AB SCIEX mgf data converter version 1.3. Subsequently, the data were searched using a Mascot server version 2.3.02 using SwissProt 2012_07 protein database (see above). Search parameters were as follows: trypsin digestion with four missed cleavages to account for the inability of trypsin to cleave at acetylated lysine residues. Trypsin specificity was set to C-terminal cleavage at lysine and arginine. Variable modifications included lysine acetylation, protein N-terminal acetylation, methionine oxidation, and conversion of glutamine to pyroglutamic acid. Carbamidomethyl cysteine was set as a fixed modification, and both precursor ion and fragment ion mass tolerances were set to 50 ppm and 0.3 Da, respectively. The maximum expectation value for accepting individual peptide ion scores [−10*Log(*p*)] was set to ≤0.01, where *p* is the probability that the observed match is a random event. For all Mascot searches, an automatic decoy search was performed choosing the *Decoy* checkbox within the search engine. For all further data processing, peptide expectation values (e-values) lower than 0.01 were chosen with all FDR rates below 0.01.
(DOCX)
