# Supplementary figures and images for: Structural, Kinetic and Proteomic Characterization of Acetyl Phosphate-Dependent Bacterial Protein Acetylation
Source: PLoS One. 2014 Apr 22;9(4):e94816. doi: 10.1371/journal.pone.0094816 (PMC3995681; doi:10.1371/journal.pone.0094816)

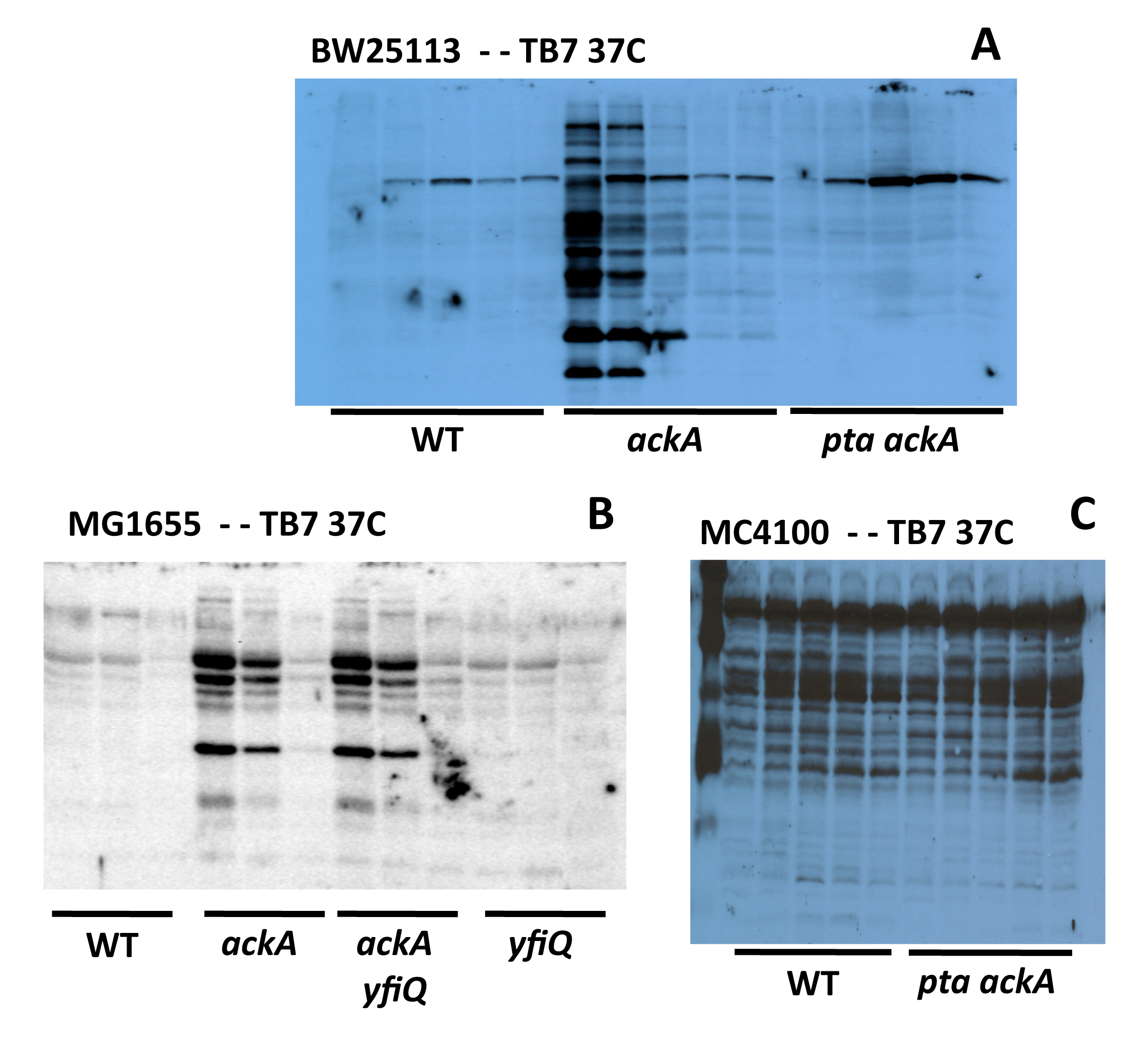

Supplement: Figure S1 — Anti-acetyllysine Western immunoblot analyses. A) E. coli WT (strain BW25113) and isogenic mutants ackA (strain AJW2921) and pta (strain AJW2922), each aerated at 37°C in TB7 and harvested at 5 time points, when the OD610 reached 0.5 or 1.0, and then at 8, 24 and 32 hours. B) E. coli WT (strain MG1655) and isogenic mutants ackA (strain AJW5052), ackA yfiQ (strain AJW5161), and yfiQ (strain AJW5035), each aerated at 37°C in TB7 and harvested at 3 time points, when the OD610 reached 0.5 or 1.0, and then at 8 hours. C) Long exposure of E. coli WT (strain PAD282) and isogenic mutant pta ackA (strain AJW2791), each aerated at 37°C in TB7 and harvested at 5 time points, when the OD610 reached 0.5 or 1.0, and then at 8, 24 and 32 hours. (TIF) [file pone.0094816.s001.tif]

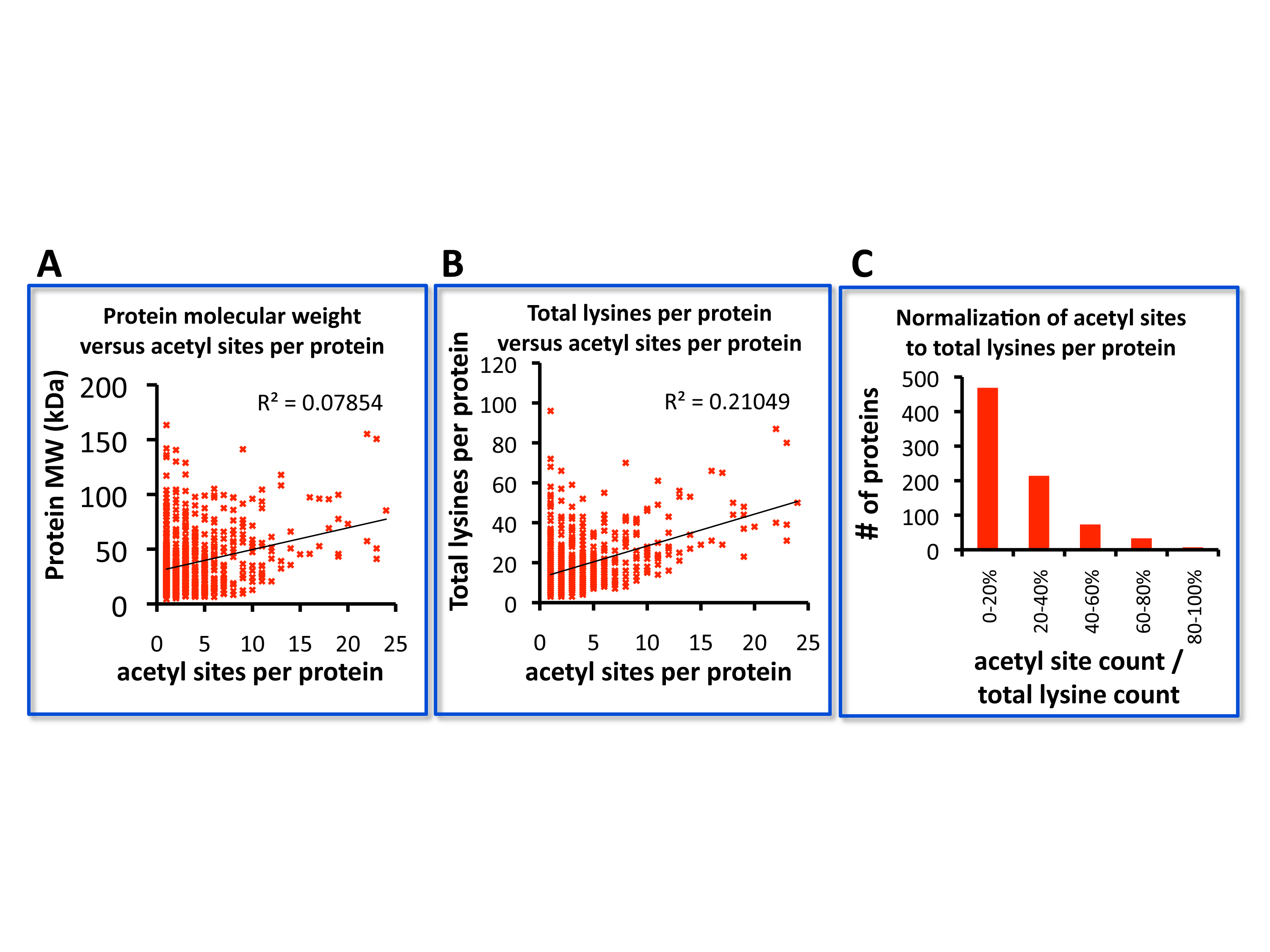

Supplement: Figure S2 — Distribution of lysine acetyl sites in E. coli proteins. Mass spectrometric analysis of WT and various mutant strains confidently identified 2730 unique lysine acetylation sites across 806 unique acetylated E. coli proteins. A) The number of identified acetyl sites per protein compared to the protein molecular weight showed no correlation. The linear regression trendline is indicated (R2 = 0.08). B) The number of identified acetyl sites per protein compared to the number of all lysine residues within a protein showed a slight correlation. The linear regression trendline is indicated (R2 = 0.21). C) Number of proteins relative to percentage of lysines acetylated (acetyl site count/total lysine count). (TIF) [file pone.0094816.s002.tif]

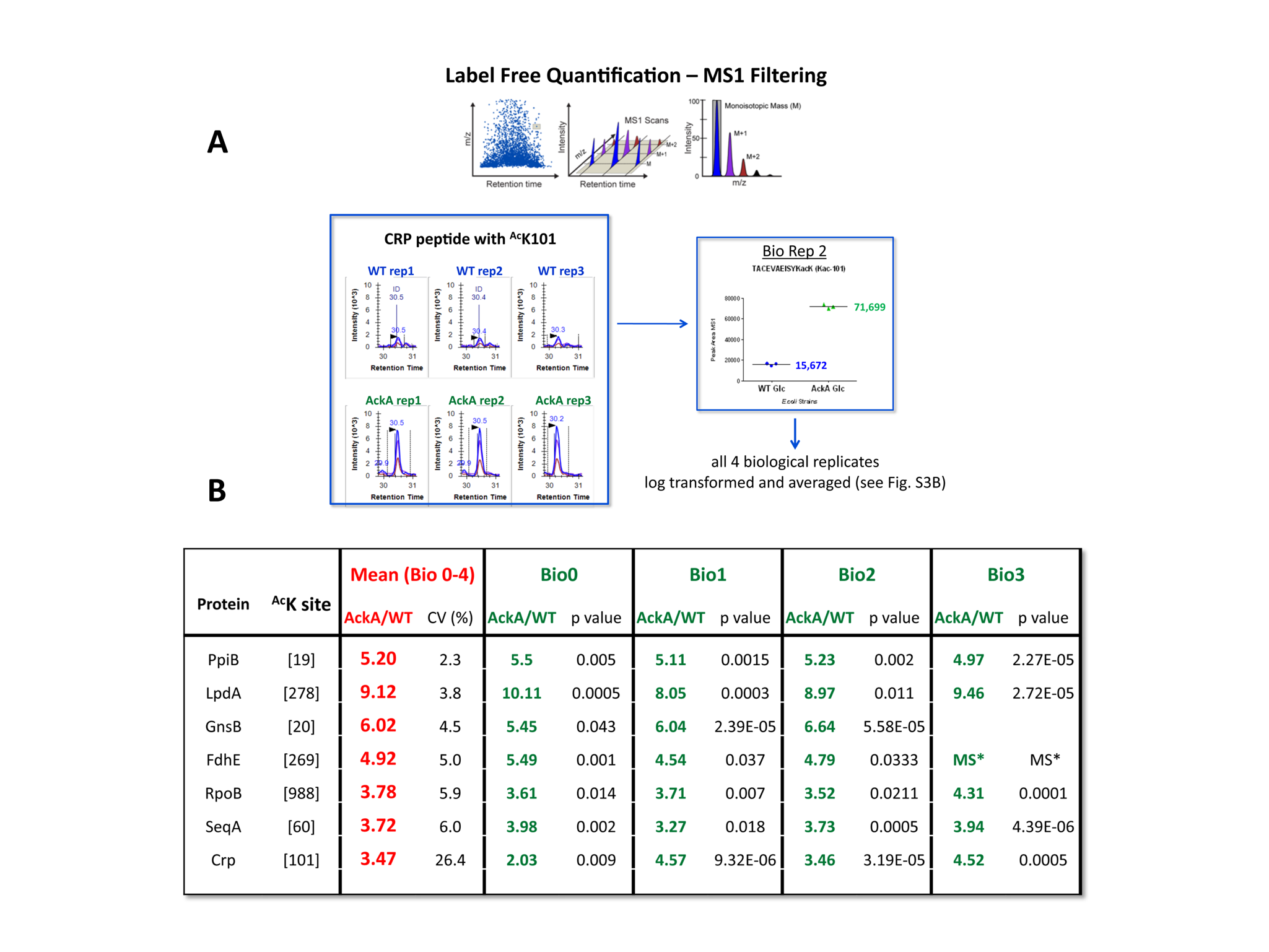

Supplement: Figure S3 — Label-free mass spectrometric quantification to assess lysine acetyl sites in E. coli . A) Skyline MS1 Filtering (Schilling, B. et al., Mol Cell Proteomics: 11, 202-214 (2012)) to quantify MS1 signal intensities of peptide precursor ions across the chromatographic gradient for the entire m/z range. For each peptide, an isotopic envelope for the molecular ion with peaks at M, M+1, and M+2 was selected showing changes in MS1 intensity over time. Extracted ion chromatograms for molecular ion isotopes were generated and the area under the curve across the peptide elution profile was integrated. An example for one (of four) biological replicate (here BioRep 2) is shown for acetylated peptide TACEVAEISYKacK with AcK101 from cAMP receptor protein (CRP) (precursor ion at m/z 720.86) for the WT and ackA mutant strains, each sample acquired in technical triplicates. Ion intensity for precursor ions for AckA MS replicates 1-3 were increased compared to WT. The integrated peak areas from BioRep 2 were then used to assess changes for acetylated lysine AcK101 and plotted for both the WT and ackA mutant strains; peak area means were determined to be 15,672 (WT) and 71,699 (ackA), revealing a 4.6 fold increase in AcK101 with a significant p-value of 9.32e−6. Biological replicates 1, 3 and 4 were processed similarly. B) To assess statistically significant regulation of acetylation in E. coli mutants relative to their WT parent, a threshold of ≥2-fold change with a p-value<0.05 was used within each biological replicate (with 3 technical MS replicates each). To be considered a ‘candidate’, significant up-regulation was required in at least 3 of the 4 independent biological replicates. To calculate mean ratios (mutant/WT) across biological replicates individual ratios were log transformed, averaged, and the resulting mean was finally transformed back into natural numbers and a coefficient of correlation (CV) was determined. Several examples are shown. Complete lists of quantifications/ [file pone.0094816.s003.tif]

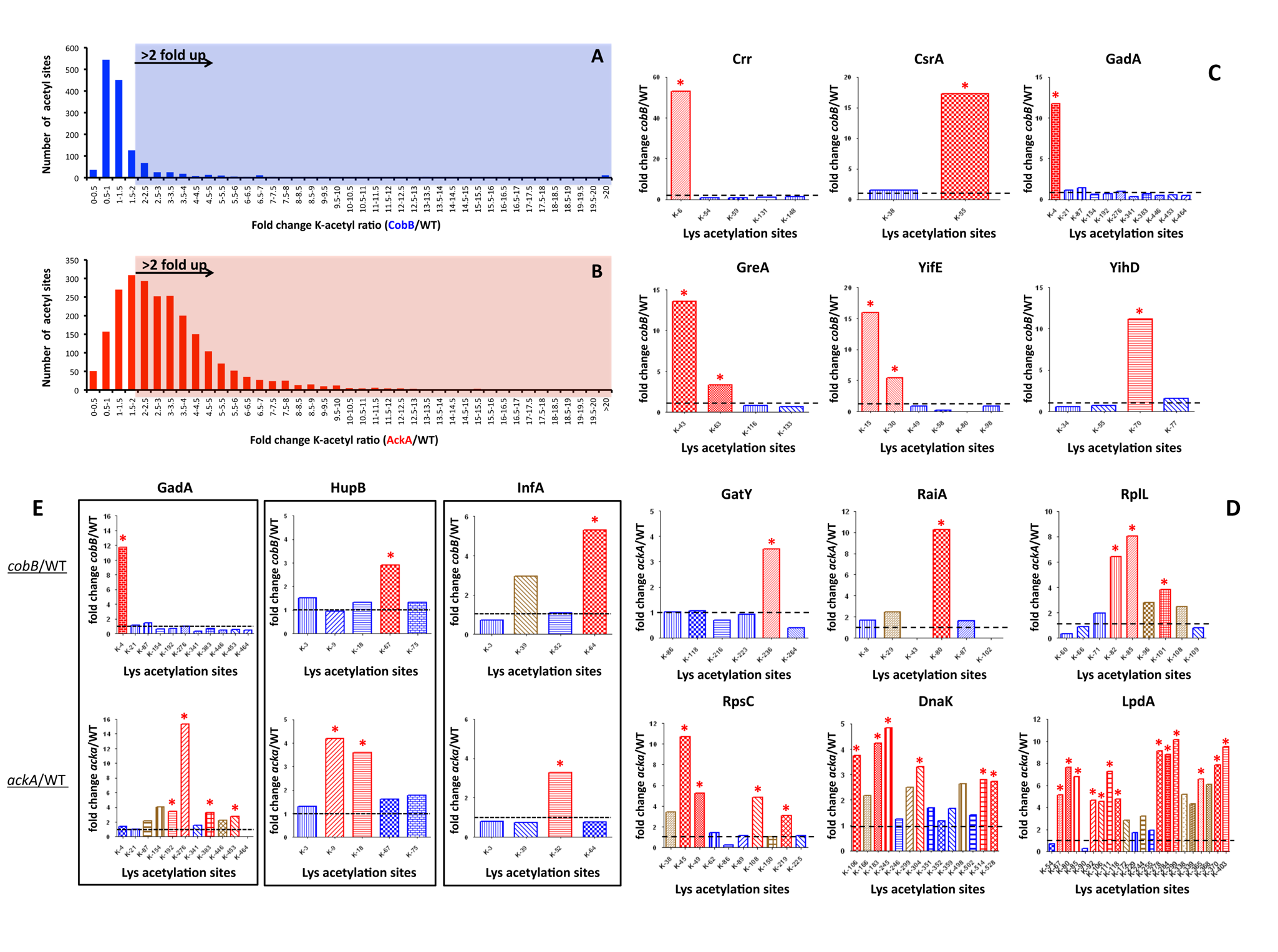

Supplement: Figure S4 — Comparison between ackA - and cobB -sensitive lysine acetyl sites. Of the 2730 unique acetylation sites from 806 proteins identified from acetyllysine-enriched fractions, quantitative mass spectrometry could be performed for 2367 quantifiable sites for the ackA mutant, and 1392 quantifiable sites for the cobB mutant. For each quantifiable site, the acetyl site ratio (mutant/WT) was calculated and the frequency of fold change for all quantifiable acetyl sites was plotted: A) cobB/WT and B) ackA/WT. For the ackA mutant (B), the fold-change profile for up-regulated acetyllysines was broader and more global than that of the cobB mutant (A), which exhibited profile changes for a smaller number of distinct sites (see Tables S15 and S16). Subsequently, further stringent statistical requirements were applied to identify statistically significant and robustly regulated acetyl sites (see Table S9). C) The acetyl site ratio (cobB/WT) for acetylated lysines in proteins Crr, CsrA, GadA, GreA, YifE, and YihD (red, >2 fold and statistically significant; blue, <2 fold). D) The acetyl site ratio (ackA/WT) for acetylated lysines in proteins GatY, RaiA, RplL, RpsC, DnaK, and LpdA (red, >2 fold and statistically significant; brown, >2 fold but not statistically significant; blue, <2 fold). E) Direct comparison of acetylated lysines from three selected proteins in the cobB mutant and the ackA mutant each relative to WT. For the proteins GadA, HupB and InfA, the acetyl site ratios (cobB/WT and ackA/WT) are presented (red, >2 fold and statistically significant; brown, >2 fold but not statistically significant; blue, <2 fold). For panels C, D, and E, the black broken line represents an acetyllysine mutant/WT peak area ratio of 1 (i.e., no change). (TIF) [file pone.0094816.s004.tif]

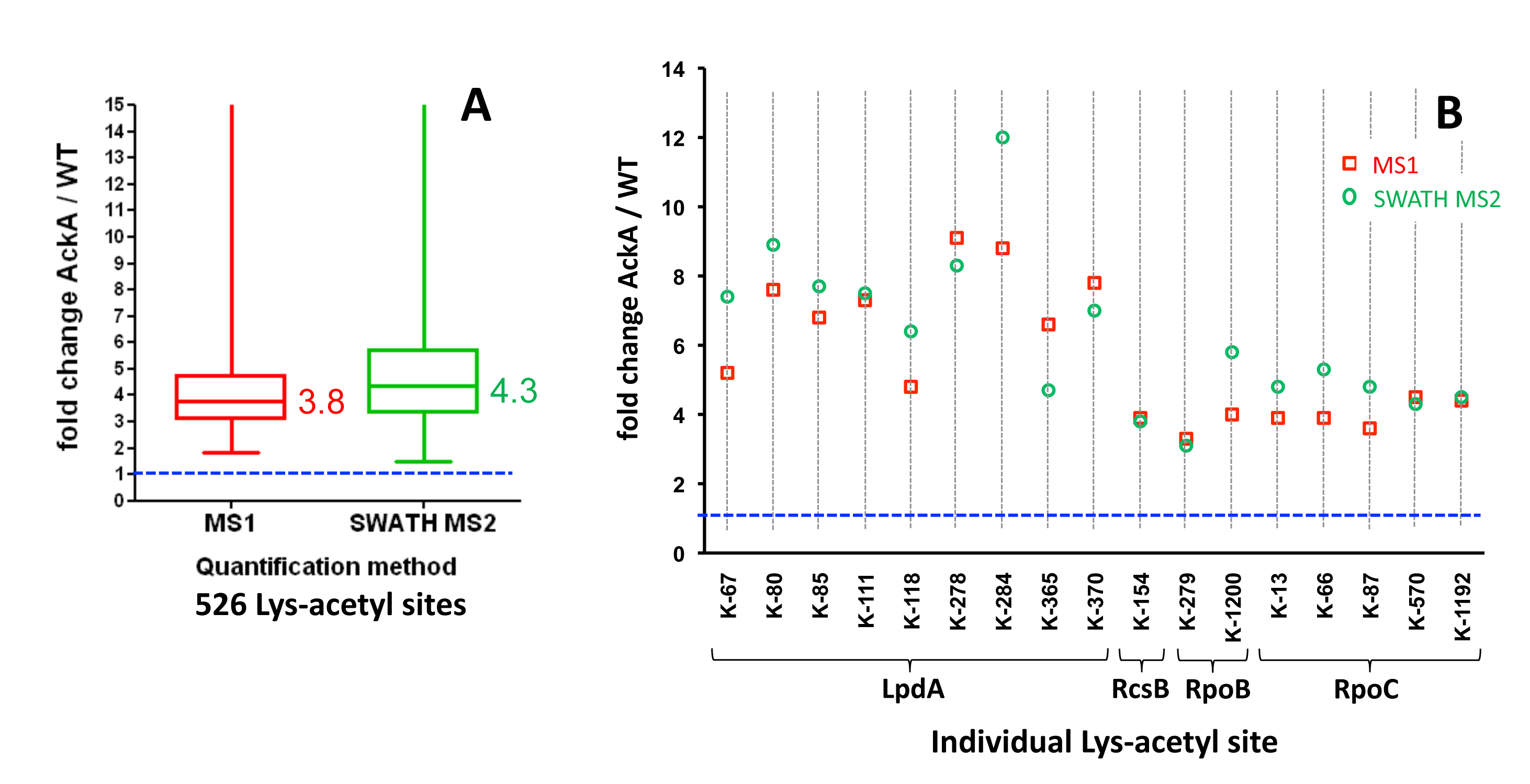

Supplement: Figure S5 — Validation by SWATH-MS2 analysis of quantification by MS1 Filtering. Acetylated lysines determined by Skyline MS1 Filtering to be robustly up-regulated in the ackA relative to WT (592 acetyl sites) were subjected to SWATH-MS2 data-independent assays for validation. A) Global comparison between MS1 Filtering (MS1 quantification) and SWATH (MS2 quantification) determining fold changes (ackA/WT) for 526 acetyllysine sites that were determined to be significant by MS1 Filtering and also could be monitored independently by SWATH acquisitions. The data points showed the following distributions for the fold change (ackA/WT) using the two different quantification approaches: median for MS1 = 3.8 and MS2 = 4.3; the 25 percentile was at 3.1 for MS1 and 3.3 for MS2, while the 75 percentile was at 4.8 for MS1 and 5.7 for MS2. B) Comparison of quantification by MS1 Filtering (red squares) and by SWATH-MS2 (green circles) for acetyl site ratios (ackA/WT) from proteins LpdA, RcsB, RpoB, and RpoC that confirm many significant acetyl site changes. For both panels (A and B), the MS1 quantification was performed on 3 technical replicates of 4 biological replicates, while the MS2 quantification was performed on 3 technical replicates of 1 biological replicate. The blue broken line represents an acetyllysine mutant/WT peak area ratio of 1 (i.e., no change). (TIF) [file pone.0094816.s005.tif]

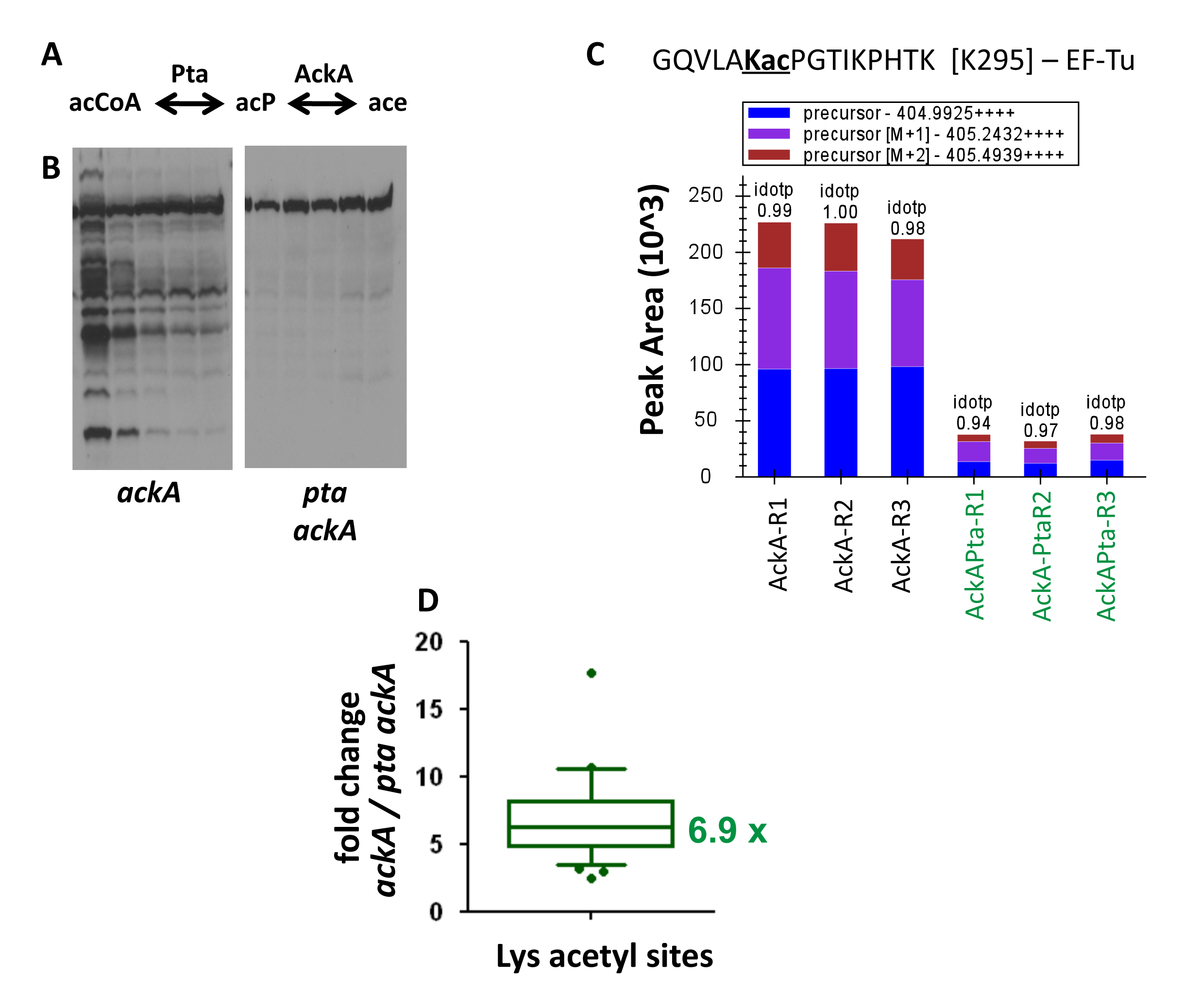

Supplement: Figure S6 — MS1 quantification of acP-dependent acetylation. A) Pta-AckA pathway schematic. B) Anti-acetyllysine antibody Western immunoblot analysis of lysates obtained from the mutants ackA (strain AJW2012) and pta ackA (strain AJW2785) grown in the absence of glucose. C) Quantitative MS1 Filtering peak area view of a selected acetylated peptide (GQVLAKacPGTIKPHTK with AcK295 from protein EF-Tu) for one selected biological replicate (out of three) with 3 technical MS replicates, comparing relative abundance of the acetylated peptide in the ackA strain relative to its abundance in the pta ackA strain (ackA/pta ackA), each grown without glucose. D) Distribution analysis of statistically significant fold changes determined for 84 acetyl sites across 3 biological replicates grown in the absence of glucose. (TIF) [file pone.0094816.s006.tif]

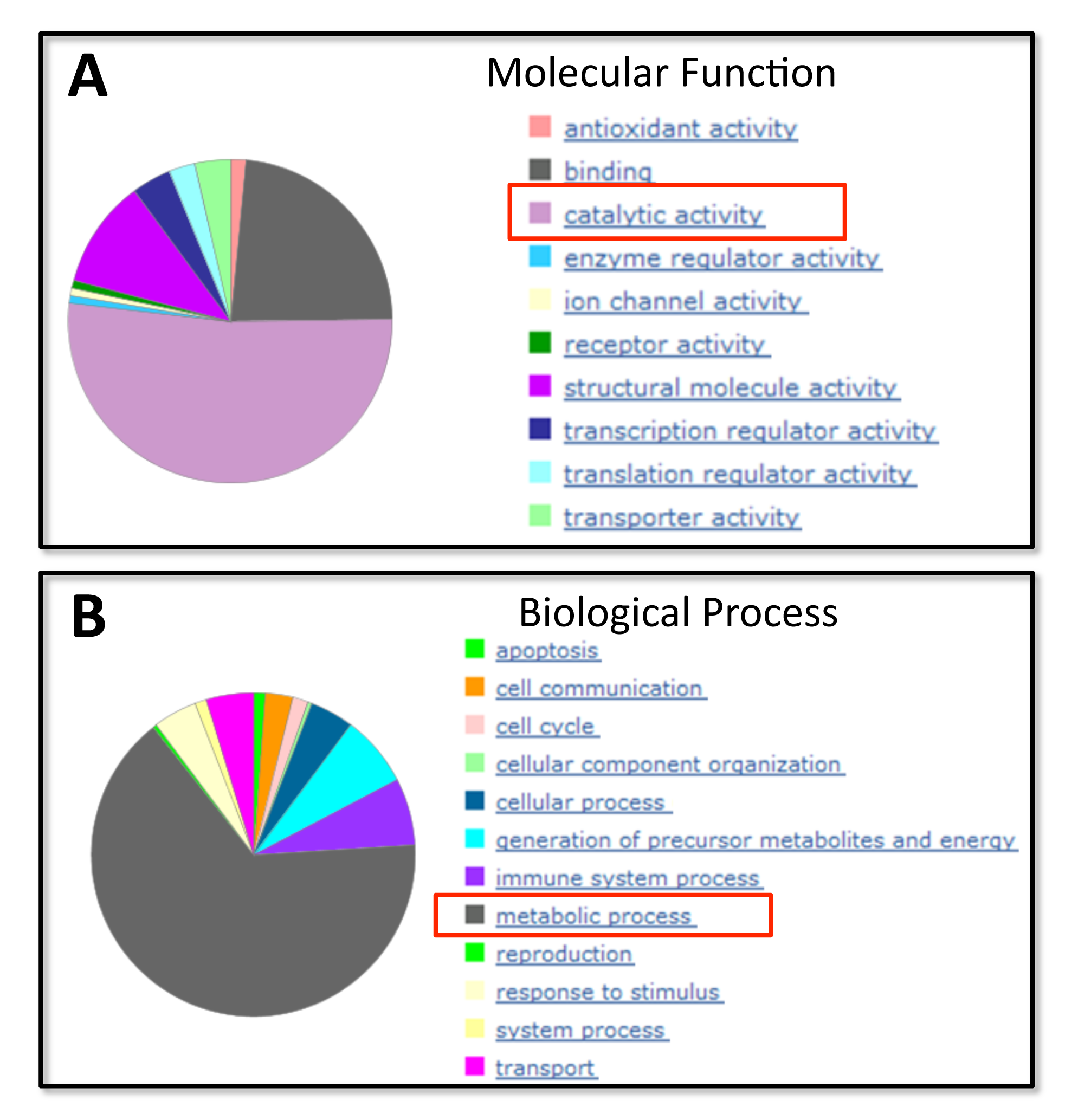

Supplement: Figure S7 — Ontology assessment of ackA -sensitive lysine acetyl sites. 292 proteins that exhibited significant up-regulation for their 592 acetyl sites in the ackA mutant relative to WT were subjected to ontology analysis using Panther. A) Molecular function GO categories, and B) Biological processes GO categories. (TIF) [file pone.0094816.s007.tif]

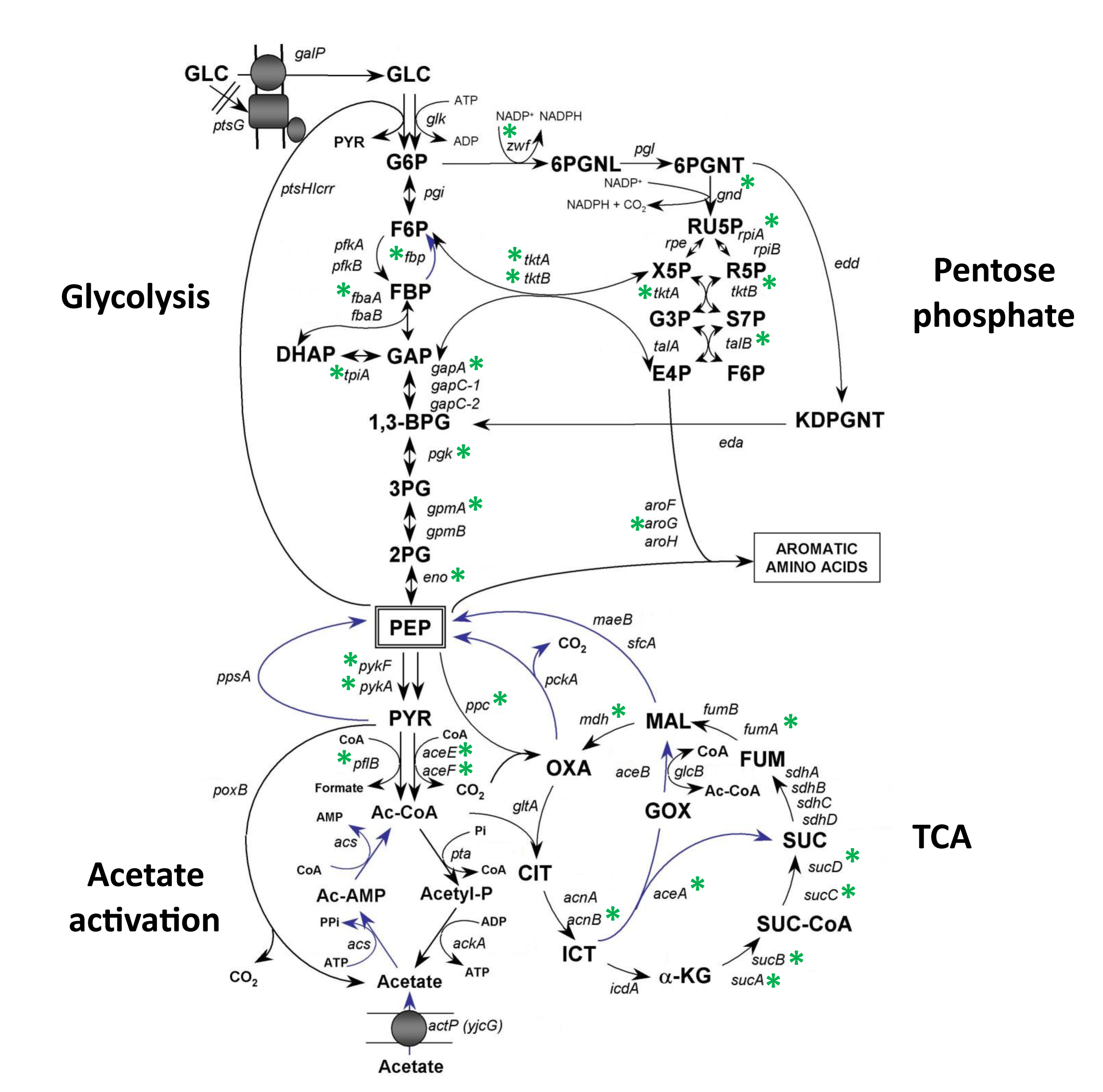

Supplement: Figure S8 — Central metabolic enzymes with ackA -sensitive lysine acetyl sites. 292 proteins that exhibited significant up-regulation for their 592 acetyl sites in the ackA mutant relative to WT were subjected to DAVID ontology and pathway enrichment analysis. Acetylated proteins that were significantly enriched for central metabolic pathways are indicated with green asterisks in the schematic (adapted from J.L. Báez-Viveros et al, Microbial Cell Factories, 2007). (TIF) [file pone.0094816.s008.tif]

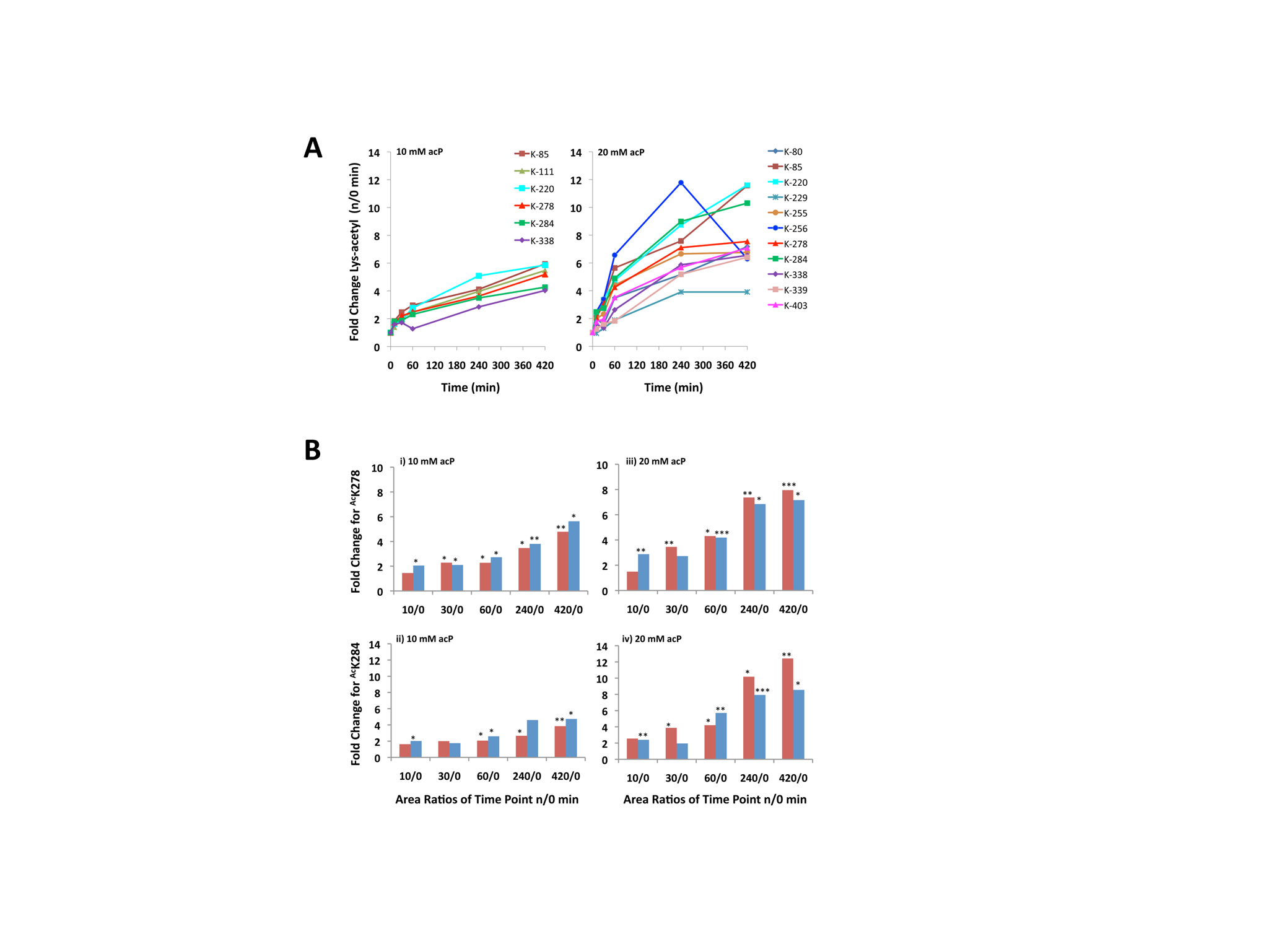

Supplement: Figure S9 — Monitoring acetylated lysine residues due to incubation of recombinant LpdA with 10 mM or 20 mM acP. A) 1.25 µM purified LpdA was incubated with either 10 mM or 20 mM acP over a defined time course. At time points 0, 5, 10, 15, and 30 min and at 1, 2, 3, 4, 5, and 7 hours, samples were subjected to quantitative MS1 Filtering analysis and relative abundance changes for each LpdA acetylation site monitored. All individual ratios were related to the zero time point (n/0 min) and were calculated from two incubation replicates (B1 and B2), each acquired in technical MS duplicates. B) Quantitative results for specific LpdA sites AcK278 (i and iii) and AcK284 (ii and iv) during incubation with 10 mM acP (i and ii) or 20 mM acP (iii and iv). For each time point, ratios (n/0 min) are shown for both incubation replicates B1 (red) and B2 (blue), each acquired in technical MS duplicates. Peptides quantified using MS1 Filtering were i) VPNGKacNLDAGK (AcK278) at 10 mM acP, ii) NLDAGKacAGVEVDDR (AcK284) at 10 mM acP, iii) VPNGKacNLDAGK (AcK278) at 20 mM acP, iv) NLDAGKacAGVEVDDR (AcK284) at 20 mM acP. (TIF) [file pone.0094816.s009.tif]

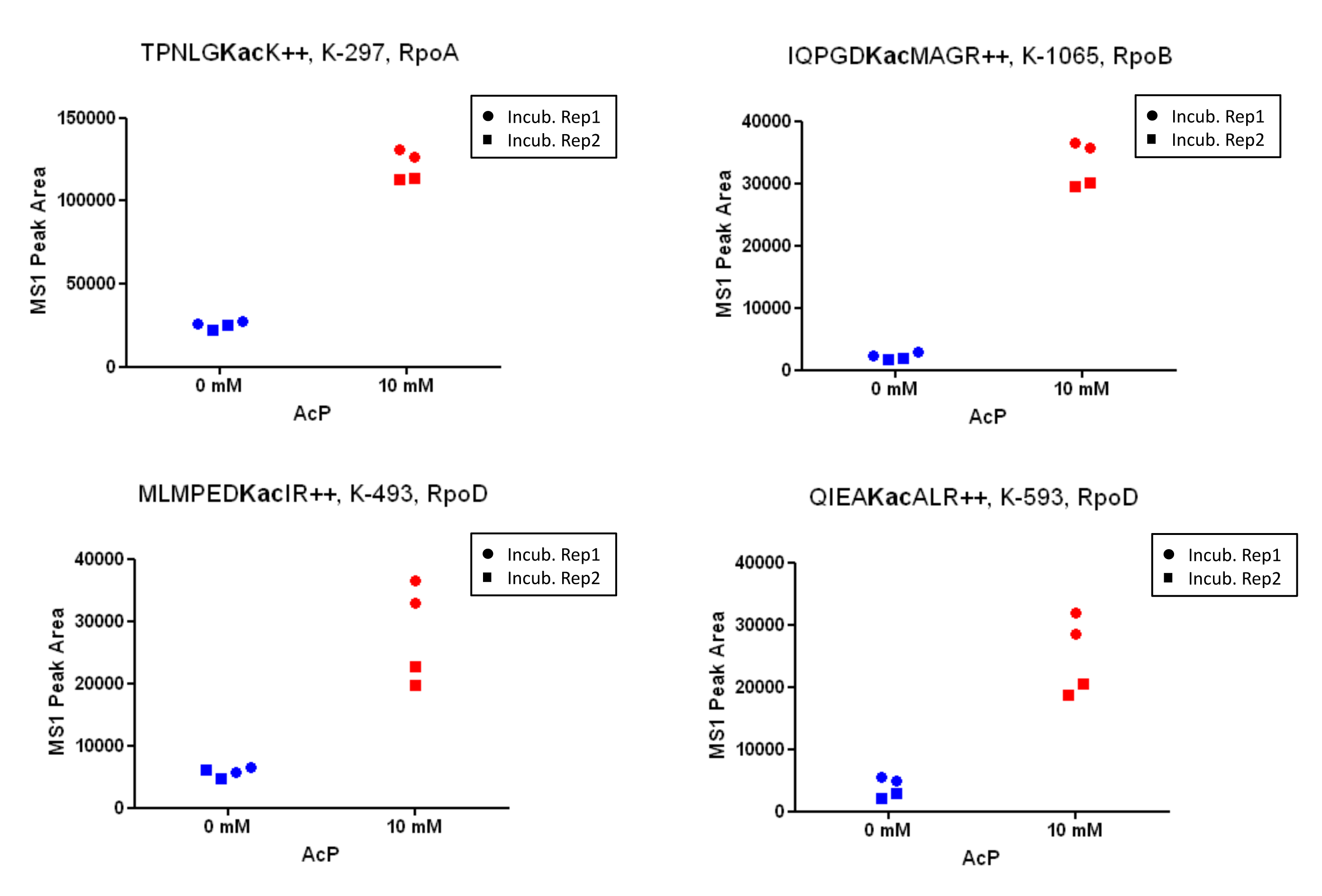

Supplement: Figure S10 — Monitoring acetylated lysine residues from incubation of purified RNAP with 10 mM acP. 1.25 µM purified RNAP was incubated with 10 mM acP for 30 min. MS1 Filtering was used to quantify relative changes over time in the abundance of acetyl sites; ratios for acetyl sites (30 min/0 min) were calculated from two incubation replicates (B1 and B2), each acquired in technical MS duplicates. Results are presented for K297 of the α subunit (RpoA, monitoring acetyl peptide TPNLGKacK), K1065 of the β subunit (RpoB, monitoring acetyl peptide IQPGDKacMAGR), and both K493 and K593 of the σ70 subunit (RpoD, monitoring acetyl peptides MLMPEDKacIR and QIEAKacALR, respectively). The following fold changes in relative acetylation abundance across both incubation and MS technical replicates were observed: 4.83-fold for AcK297 (0.5% CV); 14.84-fold for AcK1065 (4% CV); 4.71-fold for AcK493 (17% CV); and 6.71 for AcK593 (11% CV). (TIF) [file pone.0094816.s010.tif]
