# Supplementary material for: Structural, Kinetic and Proteomic Characterization of Acetyl Phosphate-Dependent Bacterial Protein Acetylation
Source: PLoS One. 2014 Apr 22;9(4):e94816. doi: 10.1371/journal.pone.0094816 (PMC3995681; doi:10.1371/journal.pone.0094816)
Supplement: Table S29 — Crystallography data collection and refinement statistics. (DOCX) [file pone.0094816.s039.docx]

|  | **tpiA native** | **tpiA AcP-treated** | **gapA AcP-treated** |
| --- | --- | --- | --- |
| ***Data collection statistics*** |  |  |  |
| Space group | P 2_1_2_1_2_1_ | P 2_1_2_1_2_1_ | P2_1_ |
| Unit cell: *a, b, c* (Å)  α,β,γ(°) | 46.07, 67.49, 149.77  90.00, 90.00, 90.00 | 46.54, 67.53, 150.34, 90.00, 90.00, 90.00 | 145.88, 69.69, 271.92,  90.00, 98.80, 90.00 |
| Wavelength (Å) | 1.03326 | 0.97856 | 0.97872 |
| Resolution (Å) | 30.00-1.80 (1.83-1.80) | 30.00-1.43 (1.45-1.43) | 30-2.85 (2.90-2.85) |
| Number of observed reflections | 44,231 (2158) | 88,659 (4,416) | 127301 (6,384) |
| R*_merge_* (%) | 8.8 (52.9) | 6.2 (53.4) | 8.6 (63.7) |
| Completeness (%) | 99.9 (98.4) | 99.9 (100.0) | 100.0 (100.0) |
| *I/σI* | 18.3, (2.6) | 22.7 (3.4) | 15.5 (2.0) |
| Phasing method | MR | MR | MR |
| ***Refinement and Validation*** |  |  |  |
| *R*_cryst_ / *R*_free_ (%) | 14.9 / 18.5 (19.4/23.6) | 13.7 / 15.6 (18.6 / 21.5) | 18.6 / 22.4 (31.4/35.9) |
| Ligands | 1 Na | 1 Cl, 1 PO4, 2 EDO, 1 PEG, 2 UVW | 12 Na, 49 Cl, 15 PO4, 6 POP, 1 PEG, 1 PGE, 1 PG4, 1UVW, 7 NAD, 1 ACT |
| Water molecules | 546 | 837 | 443 |
| Bond lengths (Å) | 0.012 | 0.012 | 0.010 |
| Bond angles (°) | 1.327 | 1.441 | 1.72 |
| Ramachandran (%) |  |  |  |
|  |  |  |  |
| Most favored regions | 93.3 | 93.5 | 88.1 |
| Additionally allowed regions | 6.7 | 6.5 | 11.6 |
| Disallowed regions | 0 | 0 | 0.3 |
| ***Acetylated lysines*** | None | None | K46 (chains a, b, c, e, f, g, i, j, k, n, o, p); K116 (chain j); K249 (chains e, i, p); K257 (chains f, h, i, j, m, o) |
| ***PDB ID*** | 4K6A | 4MVA | 4MVJ |

**Table S29.** **Crystallography data collection and refinement statistics**
