# Supplementary material for: Structural, Kinetic and Proteomic Characterization of Acetyl Phosphate-Dependent Bacterial Protein Acetylation
Source: PLoS One. 2014 Apr 22;9(4):e94816. doi: 10.1371/journal.pone.0094816 (PMC3995681; doi:10.1371/journal.pone.0094816)
Supplement: Methods S1 — Mass spectrometric and chromatographic methods. Samples were analyzed by reverse-phase HPLC-ESI-MS/MS using an Eksigent Ultra Plus nano-LC 2D HPLC system (Dublin, CA), which was directly connected to a quadrupole time-of-flight (QqTOF) TripleTOF 5600 mass spectrometer (AB SCIEX, Concord, CAN) in direct injection mode. The autosampler was operated in full injection mode overfilling a 1 µl loop with 3 µl analyte for optimal sample delivery reproducibility. Briefly, after injection, peptide mixtures were transferred onto the analytical C18-nanocapillary HPLC column (C18 Acclaim PepMap100, 75 µm I.D. ×15 cm, 3 µm particle size, 100 Å pore size, Dionex, Sunnyvale, CA) and eluted at a flow rate of 300 nL/min using the following gradient: at 5% solvent B in A (from 0-13 min), 5–35% solvent B in A (from 13–58 min), 35–80% solvent B in A (from 58–63 min), at 80% solvent B in A (from 63–66 min), with a total runtime of 90 min including mobile phase equilibration. Solvents were prepared as follows, mobile phase A: 2% acetonitrile/98% of 0.1% formic acid (v/v) in water, and mobile phase B: 98% acetonitrile/2% of 0.1% formic acid (v/v) in water. Data-dependent acquisitions (DDA): Mass spectra and tandem mass spectra were recorded in positive-ion and “high-sensitivity” mode with a resolution of ∼35,000 full-width half-maximum on the TripleTOF 5600. The nanospray needle voltage was typically 2,500 V in HPLC-MS mode. After acquisition of ∼5 to 6 samples, the MS and MS/MS spectra were calibrated during dynamic LC-MS & MS/MS autocalibration acquisitions injecting 25 fmol beta-galactosidase. For collision induced dissociation tandem mass spectrometry, the mass window for precursor ion selection of the quadrupole mass analyzer was set to ±1 m/z. The precursor ions were fragmented in a collision cell using nitrogen as the collision gas. In DDA mode, the 30 most abundant parent ions were selected for MS/MS analysis following each MS1 survey scan (approx. 50 msec per MS/MS). Dynamic exclu [file pone.0094816.s047.docx]

**Supplemental Methods S1:**

***Mass spectrometric and chromatographic methods***

Samples were analyzed by reverse-phase HPLC-ESI-MS/MS using an Eksigent Ultra Plus nano-LC 2D HPLC system (Dublin, CA), which was directly connected to a quadrupole time-of-flight (QqTOF) TripleTOF 5600 mass spectrometer (AB SCIEX, Concord, CAN) in direct injection mode. The autosampler was operated in full injection mode overfilling a 1 µl loop with 3 µl analyte for optimal sample delivery reproducibility. Briefly, after injection, peptide mixtures were transferred onto the analytical C18-nanocapillary HPLC column (C18 Acclaim PepMap100, 75 µm I.D. x 15 cm, 3 µm particle size, 100 Å pore size, Dionex, Sunnyvale, CA) and eluted at a flow rate of 300 nL/min using the following gradient: at 5% solvent B in A (from 0-13 min), 5-35% solvent B in A (from 13-58 min), 35-80% solvent B in A (from 58-63 min), at 80% solvent B in A (from 63-66 min), with a total runtime of 90 min including mobile phase equilibration. Solvents were prepared as follows, mobile phase A: 2% acetonitrile/98% of 0.1% formic acid (v/v) in water, and mobile phase B: 98% acetonitrile/2% of 0.1% formic acid (v/v) in water.

Data-dependent acquisitions (DDA): Mass spectra and tandem mass spectra were recorded in positive-ion and “high-sensitivity” mode with a resolution of ~35,000 full-width half-maximum on the TripleTOF 5600. The nanospray needle voltage was typically 2,500 V in HPLC-MS mode. After acquisition of ~ 5 to 6 samples, the MS and MS/MS spectra were calibrated during dynamic LC-MS & MS/MS autocalibration acquisitions injecting 25 fmol beta-galactosidase. For collision induced dissociation tandem mass spectrometry, the mass window for precursor ion selection of the quadrupole mass analyzer was set to ± 1 *m/z*. The precursor ions were fragmented in a collision cell using nitrogen as the collision gas. In DDA mode, the 30 most abundant parent ions were selected for MS/MS analysis following each MS1 survey scan (approx. 50 msec per MS/MS). Dynamic exclusion features were based on value M not *m/z* and were set to an exclusion mass width 50 mDa and an exclusion duration of 30 sec.

Data-independent acquisitions (DIA), MS/MS^ALL^ with SWATH acquisitions: In the SWATH-MS2 acquisition, a Q1 window of 25 *m/z* was selected to cover the mass range of m/z 400-1000 in 24 segments (24 x 100 msec), yielding a cycle time of 3.25 sec, which includes one 250 msec MS1 scan. SWATH-MS2 produces complex MS/MS spectra, which are a composite of all the analytes within each selected Q1 *m/z* window.

***Bioinformatic database searches***

Mass spectrometric data was analyzed using ProteinPilot (Shilov, I.V. et al. Mol. Cell. Proteomics: 6, 1638-1655, **2007**) (AB SCIEX Beta 4.5, revision 1656) and the Paragon algorithm (4.5.0.0,1654). The following sample parameters were used: trypsin digestion, cysteine alkylation set to iodoacetamide, urea denaturation, acetylation emphasis, and species *E. coli*. Trypsin specificity was set at C-terminal lysine and arginine. Processing parameters were set to "Biological modification" and a thorough ID search effort was used. During the search, ProteinPilot performs an automatic mass recalibration of the data sets based on highly confident peptide spectra. Specifically, a first search iteration is done to select high confidence peptide identifications that are used to recalibrate both the MS and MS/MS data, which is automatically re-searched. All data files were searched using the SwissProt 2012_07 database) with a total of 8870 *E. coli* protein sequences. For ProteinPilot Searches, to assess and restrict rates of false positive peptide/protein identifications, we used the Proteomics System Performance Evaluation Pipeline (PSPEP) tool available in ProteinPilot 4.5 beta. This tool automatically creates a concatenated forward and reverse decoy database, and provides an Excel file output of the experimentally determined false discovery rate at the spectral, peptide and protein levels with standard statistical errors. The discriminating variable for the Paragon Algorithm is the peptide confidence value, which is a 0-99 scaled real number (Shilov, I.V. et al. Mol. Cell. Proteomics: 6, 1638-1655, **2007**). For database searches, a cut-off peptide confidence value of 99 was chosen with the following justification. For searching the databases, the Protein Pilot false discovery rate (FDR) analysis tool (PSPEP) algorithm (Shilov, I. V. et al. Mol. Cell. Proteomics: 6, 1638-1655, **2007**) provided a global FDR of 1% and a local FDR at 1% in all cases.

Mass spectral data sets also were analyzed and searched using Mascot. In Mascot (Perkins, D.N. et al., Electrophoresis 20, 3551-3567, **1999**), peak lists were generated using the AB SCIEX mgf data converter version 1.3. Subsequently, the data were searched using a Mascot server version 2.3.02 using SwissProt 2012_07 protein database (see above). Search parameters were as follows: trypsin digestion with four missed cleavages to account for the inability of trypsin to cleave at acetylated lysine residues. Trypsin specificity was set to C-terminal cleavage at lysine and arginine. Variable modifications included lysine acetylation, protein N-terminal acetylation, methionine oxidation, and conversion of glutamine to pyroglutamic acid. Carbamidomethyl cysteine was set as a fixed modification, and both precursor ion and fragment ion mass tolerances were set to 50 ppm and 0.3 Da, respectively. The maximum expectation value for accepting individual peptide ion scores [-10*Log(*p*)] was set to ≤0.01, where *p* is the probability that the observed match is a random event. For all Mascot searches, an automatic decoy search was performed choosing the *Decoy* checkbox within the search engine. For all further data processing, peptide expectation values (e-values) lower than 0.01 were chosen with all FDR rates below 0.01.
